# Cellular iron depletion enhances behavioral rhythm by limiting brain Per1 expression in mice

**DOI:** 10.1111/cns.14592

**Published:** 2024-02-22

**Authors:** Qiong Wu, Qiuyang Ren, Xin Wang, Huiyuan Bai, Dandan Tian, Guofen Gao, Fudi Wang, Peng Yu, Yan‐Zhong Chang

**Affiliations:** ^1^ Laboratory of Molecular Iron Metabolism, Key Laboratory of Molecular and Cellular Biology of Ministry of Education, Hebei Key Laboratory of Animal Physiology, Biochemistry and Molecular Biology, Hebei Collaborative Innovation Center for Eco‐Environment, Hebei Research Center of the Basic Discipline of Cell Biology, College of Life Sciences Hebei Normal University Shijiazhuang China; ^2^ Hebei Key Laboratory of Chinese Medicine Research on Cardio‐Cerebrovascular Disease, College of Basic Medicine Hebei University of Chinese Medicine Shijiazhuang Hebei Province China; ^3^ School of Public Health Zhejiang University School of Medicine Hangzhou Zhejiang China

**Keywords:** aging, circadian rhythm, clock genes, iron, Per1

## Abstract

**Aims:**

Disturbances in the circadian rhythm are positively correlated with the processes of aging and related neurodegenerative diseases, which are also associated with brain iron accumulation. However, the role of brain iron in regulating the biological rhythm is poorly understood. In this study, we investigated the impact of brain iron levels on the spontaneous locomotor activity of mice with altered brain iron levels and further explored the potential mechanisms governing these effects in vitro.

**Results:**

Our results revealed that conditional knockout of *ferroportin 1* (*Fpn1*) in cerebral microvascular endothelial cells led to brain iron deficiency, subsequently resulting in enhanced locomotor activity and increased expression of clock genes, including the circadian locomotor output cycles kaput protein (Clock) and brain and muscle ARNT‐like 1 (Bmal1). Concomitantly, the levels of period circadian regulator 1 (PER1), which functions as a transcriptional repressor in regulating biological rhythm, were decreased. Conversely, the elevated brain iron levels in APP/PS1 mice inhibited autonomous rhythmic activity. Additionally, our findings demonstrate a significant decrease in serum melatonin levels in *Fpn1* ^*cdh5*^ ‐CKO mice compared with the *Fpn1* ^flox/flox^ group. In contrast, APP/PS1 mice with brain iron deposition exhibited higher serum melatonin levels than the WT group. Furthermore, in the human glioma cell line, U251, we observed reduced PER1 expression upon iron limitation by deferoxamine (DFO; iron chelator) or endogenous overexpression of FPN1. When U251 cells were made iron‐replete by supplementation with ferric ammonium citrate (FAC) or increased iron import through transferrin receptor 1 (TfR1) overexpression, PER1 protein levels were increased. Additionally, we obtained similar results to U251 cells in mouse cerebellar astrocytes (MA‐c), where we collected cells at different time points to investigate the rhythmic expression of core clock genes and the impact of DFO or FAC treatment on PER1 protein levels.

**Conclusion:**

These findings collectively suggest that altered iron levels influence the circadian rhythm by regulating PER1 expression and thereby modulating the molecular circadian clock. In conclusion, our study identifies the regulation of brain iron levels as a potential new target for treating age‐related disruptions in the circadian rhythm.

## Graphical abstract

The present study indicates that changes in brain iron levels can regulate thythmic activities of mice through modifying Per1 expression and thus offers brain iron levels as an innovative target to treat age‐associated dysfunction of the circadian rhythm.

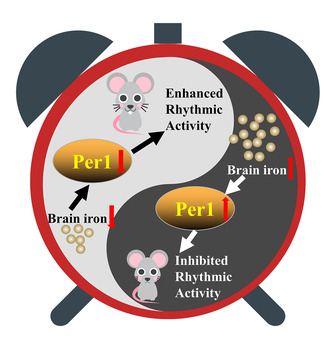

## 1 INTRODUCTION

The circadian clock, which is mainly controlled by the suprachiasmatic nucleus (SCN) of the hypothalamus, coordinates the internal functions of the organism within 24‐h, self‐perpetuating oscillations.^1^ The circadian system possesses crucial roles in most physiological processes, including sleep alertness and various areas of cognitive performance. Circadian misalignment is widespread in the elderly, who often exhibit declined sleep quality. Furthermore, there is an accumulation of misalignment with age since the circadian rhythm cannot be completely resorted by recovery sleep.^2^, ^3^ Additionally, aging‐related neurodegenerative diseases, which are usually accompanied by cognitive impairment, can also disrupt rhythmic oscillations.^4^ Importantly, several studies have shown that perturbation of regular biological rhythms, linked to the pathologies of diseases such as Alzheimer's disease (AD), ischemic stroke, other mental disorders, and cancer,^4^, ^5^, ^6^, ^7^, ^8^ results in numerous physiological disorders in organismal homeostasis, ultimately affecting quality of life.^9^

Iron is an essential participant and regulator of various fundamental physiological activities.^10^ Several studies have demonstrated that aging is accompanied by elevated cellular iron deposition. Abnormal iron accumulation and the consequential induction of redox stress have been widely reported to participate in the pathogenesis of aging‐related neurodegenerative diseases.^11^, ^12^, ^13^ At the same time, disruptions in circadian rhythms have also been increasingly recognized as symptoms of aging and age‐associated neurodegenerative disorders, including AD.^14^, ^15^ Clinical and animal model studies have further revealed the presence of progressive circadian dysfunction throughout the course of these diseases.^16^, ^17^ However, few studies have investigated the effects of iron or brain iron levels on circadian rhythm maintenance. Liu et al. reported that iron accumulation with age altered the metabolic pattern and expression of circadian clock genes through decreased histone methylation in the mouse liver.^18^ Additionally, iron‐induced redox toxicity may also lead to a disturbance in the biological circadian clock. A recent review by T. Ishii et al. discusses how circadian rhythm‐induced improvements in the expression of an important antioxidant pathway, brain‐derived neurotrophic factor (BDNF)‐Nrf2 in astrocytes, can strengthen astrocyte–neuron interactions and protect dopaminergic neurons from iron‐mediated cell death (ferroptosis) and redox toxicity in rodent brains.^19^ Thus, it is important to understand whether alterations in iron status can alter biological rhythms and affect the expression of circadian clock genes.

In the present study, we investigated the influence of altered brain iron levels on locomotor activity and circadian rhythms in 10‐month‐old mice. Mice with conditional knockout of the cellular iron exporter, FPN1, in microvascular endothelial cells (*Fpn1* ^*Cdh5*^‐cKO) were used in this study since they display low brain iron levels.^20^ We also used APP/PS1 double‐transgenic mice that are reported to exhibit brain iron accumulation with age.^21^ The *Fpn1* ^*Cdh5*^‐cKO mice exhibited increased locomotor activity, while the APP/PS1 mice displayed inhibited rhythmic activities. Our initial results suggest that mice with altered brain iron levels possess altered behavioral rhythms. We proceeded to investigate the regulation of the transcriptional/translational feedback loop (TTFL) comprising the clock genes and their corresponding proteins.^22^, ^23^ The TTFL is responsible for maintaining the oscillation system and synchronizing the cycle with the environment. In TTFL regulation, the core clock gene, PER1, acts as a transcriptional repressor, while CLOCK functions as an enhancer that can promote rhythmic activities.^24^, ^25^ We found that *Fpn1* ^*Cdh5*^‐cKO mice with declined brain iron levels exhibit decreased expression of Per1, with elevated CLOCK levels. These results imply that alterations in brain iron levels can indeed alter circadian clock gene expression and lead to modified TTFL regulation. Finally, we investigated the potential molecular mechanisms involved in the effects of iron on clock gene regulation in U251 cells. We conclude that iron can modulate the activity of the PER1‐CLOCK‐BMAL1 regulatory pathway. We propose rhythmic regulation of brain iron levels as a novel target to improve age‐related disruption of the circadian rhythm.

## 2 MATERIALS AND METHODS

### 2.1 Animals

Ten‐month‐old wild‐type (WT) mice, APP/PS1 transgenic mice, *Fpn1* ^flox/flox^ mice, and *Fpn1* ^*Cdh5*^‐cKO mice were used in this study. The APP/PS1 double‐transgenic mice and WT control mice are in the C57BL/6J background and were purchased from Beijing Zhongke Zesheng Technology Co., Ltd (Beijing, China). The APP/PS1 mice were crossed with WT mice, and the genotypes of the offspring were previously determined.^21^ The VE‐Cadherin‐Cre (Cdh5‐Cre) transgenic mice on a C57BL/6 genetic background used in the study were a generous gift from Dr. Xiao Yang of the Beijing Institute of Life Omics, and were characterized in previous study.^26^ In the present study, we generated *Fpn1* ^*Cdh5*^‐cKO mice by mating Cdh5‐Cre mice with *Fpn*1^flox/flox^ mice on a C57BL/6 background. The genotypes of the Cdh5‐Cre and *Fpn*1^flox/flox^ mice were described previously.^20^ All the mice were bred and maintained in a specific pathogen‐free (SPF) animal facility under a 12 h light and 12 h dark cycle. Mice were allowed free access to water and food and were randomly assigned to experimental groups. All procedures were carried out in accordance with the National Institutes of Health Guide for the Care and Use of Laboratory Animals and approved by the Animals Ethics Committee of Hebei Normal University, China.

### 2.2 Experimental design and measurement of rodent locomotor activity

Male mice were used in all experiments. The locomotor activities of 10‐month‐old mice were analyzed (*n* = 3–4 per group). The recordings of locomotor activity spanned 8 consecutive days, including 6 days of normal light/dark cycles (LD, 12:12, with lights on at 8:00 a.m. = zeitgeber time (ZT) 00) and 2 days of dark/dark (DD) cycles. The animals were acclimated to the experimental conditions for at least 5 days prior to the experiments. Individual mice were housed in cages equipped with infrared movement detectors connected to an automated recording system (the Mouse Wheel Running Activity Monitoring System, ZS‐Labmaze IV, Beijing Zhongshi Dichuang Technology Development Co., Ltd., Beijing, China). Spontaneous locomotor activity was continuously recorded throughout the entire experiment and analyzed using Trigger Master software (v4.1.0, Beijing Zhongshi Dichuang Technology Development Co., Ltd., Beijing, China).

### 2.3 Cell culture and treatment

U251 cells and MA‐c cells were maintained in DMEM with high glucose, supplemented with 10% fetal calf serum and 100 U/mL penicillin/streptomycin at 37°C and 5% CO_2_. Overexpression and knockdown of Per1 were achieved by transfecting pcDNA3.1‐Per1 plasmid and si‐Per1, respectively. The full‐length coding sequence (CDS) of Per1 (NM_002616.3) was inserted into the vector. The shRNAs (shRNA1, 2, and 3) for Per1 were designed and synthesized by Youbio Technology, China. For cellular transfection, U251 cells were seeded in six‐well plates, and grown to 90% confluence before transfection. The cells were transiently transfected with pcDNA3.1 (empty vector, EV, 2500 ng), PER1 overexpression plasmid (pcDNA3.1‐*PER1*, 2500 ng), or the shRNAs (shRNA1, 2, and 3) for *PER1* (2500 ng). Transient transfection was performed using Lipofectamine 3000 (#MAN0009872, Invitrogen) according to the manufacturer's instructions.

The duration of treatment of U251 and MA‐c cells with DFO or FAC was 24 h. To investigate the impact of collecting cells at different time points on the expression of circadian clock proteins, we used two time points. In the first approach, DFO or FAC was added to the cells at 10:00 p.m. on the first day. After 24 h (the following day at 10:00 p.m.), DFO or FAC was removed, and dexamethasone was added to the culture medium for 2 h (until midnight, 12:00 p.m.). On the morning of the third day at 8:00 a.m., the cells were harvested and immediately processed for subsequent studies. In the second dosing approach, the drug administration was the same as the first method, but the initial addition of DFO or FAC was performed at 10:00 a.m. Consequently, the collection and processing of cells were performed at 8:00 p.m. on the second day.

For the other cells in this study, we applied dexamethasone at 10:00 p.m. on the first day to induce synchronization, removed dexamethasone after 2 h, and collected the cells at 8:00 a.m. on the second day. These treatments help ensure that the cells are in a synchronized state, which is beneficial for studying the impact of circadian clock protein expression.

### 2.4 Preparation of human glioma U251 cells overexpressing FPN1 or TfR1

The full‐length CDS of the target gene, *FPN1* (NM_014585.6) or *TFR1* (NM_001128148.3), was inserted into a lentivirus vector that confers Puro resistance and is regulated by the TetOne operator (pLVX‐TetOne‐puro‐SLC40A1 and pLVX‐TetOne‐puro‐TFRC). The virus was generated by transfection into the HEK293T cells. The modified lentivirus was then prepared to infect U251 cells. After drug resistance screening, clones were selected and expanded for culture to establish U251 cell lines stably overexpressing FPN1 (U251‐FPN1) or TfR1 (U251‐TfR1). The U251‐FPN1 and U251‐TfR1 cells required induction with 100 nM doxycycline for 2 h to overexpress the respective proteins prior to additional treatments.

### 2.5 MTT assay

The MTT assay was used to evaluate cell viability. After treatment with a gradient of FAC (#F5879, Sigma) or DFO (#D9533, Sigma), the culture medium was discarded, MTT (0.5 mg/mL, #M8180, Solarbio) was added, and the cells were incubated at 37°C in a 5% CO_2_ incubator for 4 h. DMSO (150 μL, #D8371, Solarbio) was then added to dissolve the formazan, and the absorbance at 575 nm was measured using a microplate reader (Synergy H4, Bio‐Tek, USA).

### 2.6 Enzyme‐linked immunosorbent assay (ELISA)

Mouse serum levels of melatonin were measured using a commercial ELISA kit (E‐EL‐M0788c, Elabscience, Wuhan, China). All experimental steps were performed at room temperature and in duplicate. Samples were quantified by spectrophotometry at 450 nm.

### 2.7 Immunohistochemistry (IHC) and double immunofluorescence (double‐IF)

We used 15‐μm‐thick, frozen brain slices containing the regions selected for investigation. The brain slices were washed three times in 0.01 M cold PBS and then incubated in 3% H_2_O_2_ for 10 min to quench endogenous peroxidase activity. Antigen retrieval was carried out in microwave oven in 0.01 M citric acid solution (pH 6) for 15 min. After washing with PBS, the sections were blocked in goat serum for 70 min at room temperature. Primary antibodies, including Ferritin‐H (FtH, 1:400, #ab183781, Abcam), Ferritin‐L (Ft‐L, 1:200, #ab109373, Abcam), Per1 (1:100, #abs118499, Absin), or Clock (1:70, #5157 s, Cell Signaling Technology), were added at 4°C overnight. For IHC staining, the sections were then incubated with biotin‐labeled goat anti‐rabbit serum for 1 h at 37°C after washing with PBS. Next, the brain slides were treated with an avidin‐biotinylated horseradish peroxidase complex for 1 h at 37°C and stained using a DAB kit, followed by dehydration with an alcohol gradient, treatment with xylene, and sealing in adhesive resin. Images of the brain sections were acquired using an Axio Imager Z2 (Zeiss) attached to a tissue cytometer Tissue FAXS (TissueGnostics, Vienna, Austria). For double IF detection, after washing with PBS, the corresponding secondary antibodies, including DyLight 488 (1:200, #A23210, Abbkine) and DyLight 549 (1:200, #23320, Abbkine), were added at 37°C for 1 h, after which the sections were stained with DAPI (1:1000, #62248, Thermo Fisher). For double‐IF experiments, the brain sections were scanned using an Olympus FV3000 confocal laser scanning microscope. The images were analyzed using imageJ software (National Institutes of Health).

### 2.8 RNA isolation and quantitative real‐time PCR (qRT‐PCR) analysis

The total RNA from U251 cells or MA‐c cells was extracted using TRIzol reagent, following the manufacturer's instructions. Total RNA (1 μg) was reverse transcribed using MMLV reverse transcriptase with oligo‐dT primers according to the manufacturer's instructions (#639574, Takara Bio, China). SYBR Green PCR Master Mix (#CW0659, CoWin Biosciences, China) was used for PCR amplification. The cycle time (Ct) values for the genes of interest were normalized to those of β‐actin mRNA. The data were analyzed using the ΔΔCt method. The primer sequences used for amplification were as follows:

**Table:** 

*BMAL1*: forward‐GGATGTGACCGAGGGAAGAT; reverse‐ CGTCGTGCTCCAGAACATAAT
*PER1*: forward‐ AACGGGATGTGTTTCGGGGTGC; reverse‐ AGGACCTCCTCTGATTCGGCAG
*CLOCK*: forward‐ GGGTTGGTGGAAGAAGATGA; reverse‐ AGCATTGTGGAAGAAGATGA
*CRY1*: forward‐ CACTGGTTCCGAAAGGGACTC; reverse‐ CTGAAGCAAAAATCGCCACCT
*ACTB*: forward‐AAGGGACTTCCTGTAACAATGCA; reverse‐CTGGAACGGTGAAGGTGACA.

### 2.9 Western blot analysis

We previously described a method for protein extraction from various mouse brain regions (cortex and hippocampus).^27^ For cellular proteins, we directly added RIPA buffer containing 1% NP40 and protease inhibitor cocktail tablets into the cell suspension to lyse the cells. After centrifugation, the supernatant was collected and the total protein concentration was measured using a BCA protein quantification kit (#20201ES76, Yeasen Biotechnology, Shanghai, China). About 20–40 μg protein lysate was resolved by SDS‐PAGE. The protein was then transferred to a nitrocellulose membrane, which was blocked in 5% nonfat milk dissolved in TBS‐T for 1.5 h at room temperature. The desired primary antibodies FtL (1:5000, Abcam), FtH (1:5000, Abcam), FPN1 (1:10,000, #MTP11‐S, ADI), TfR1 (1:10,000, #13‐6890, Abcam), PER1 (1:10,000, Absin, abs118499), Per2 (1:20,000, #67513‐1‐1 g, Proteintech Group), CLOCK (1:3000, Cell Signaling Technology), BMAL1 (1:8000, #14020S, Cell Signaling Technology), p‐GSK3β (1:5000, #9336, Cell Signaling Technology), or GSK‐3α/β (1:5000, #sc‐7219, Santa Cruz) were then added at 4°C overnight. The membrane was washed with TBS‐T, and then incubated with anti‐rabbit or anti‐mouse IgG secondary antibodies conjugated with horseradish peroxide for 90 min at room temperature. After washing again with TBS‐T, immunoreactive proteins were detected using the super ECL detection reagent kit (#36208ES60, Yeasen Biotechnology, Shanghai, China) and visualized using a chemiluminescence imager (Fujifilm Las‐4000, Fujifilm, Japan). The relative band intensities of the proteins were quantified using imageJ software and are presented as the ratio of each to β‐actin.

### 2.10 Statistical analysis

The data are expressed as the mean ± standard error of mean (SEM), as specifically indicated in the figure legends. The datasets were tested for normality distribution using the Shapiro–Wilk test using SPSS 21.0 software; those that passed the normality test were advanced to statistical analysis. The data were analyzed using a two‐tailed Student's *t*‐test for independent datasets and as specifically described in the figure legends. The data were analyzed using Prism v9.0 (GraphPad Software). *p* values <0.05 were considered statistically significant, while *p* values <0.01 were considered remarkably significant. Data from this study are available from the corresponding authors, Yan‐Zhong Chang and Peng Yu, upon reasonable request.

## 3 RESULTS

### 3.1 Altered brain iron levels results in shifts in locomotor activity rhythm

**FIGURE 1:**
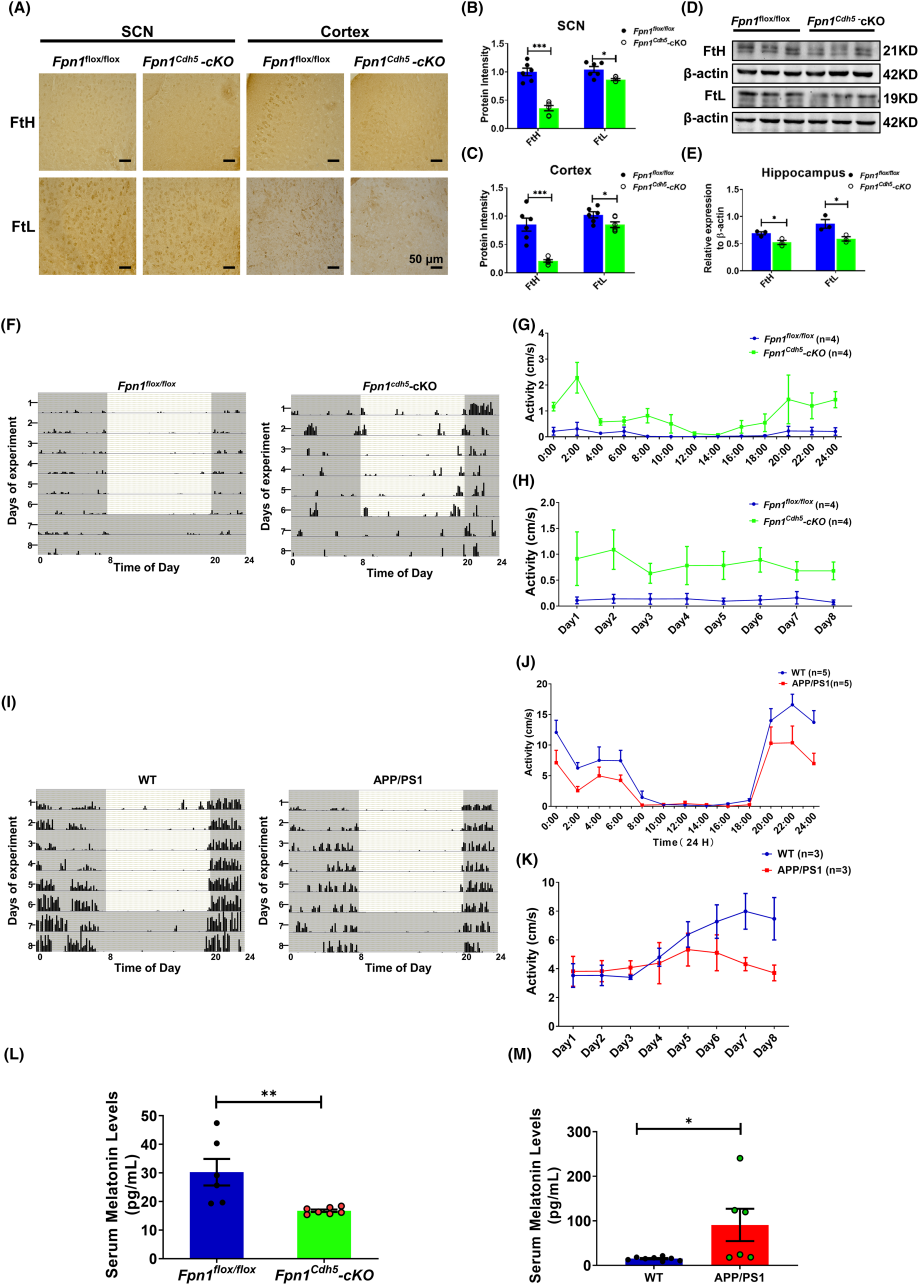
Altered expression of FtH and FtL in *Fpn1* ^*Cdh5*^‐cKO mice and the locomotor activity of *Fpn1* ^*Cdh5*^‐cKO and APP/PS mice. The distribution and expression of FtH and FtL were detected by immunohistochemistry (IHC); the representative IHC results of FtH and FtL staining in the SCN and cortex regions are shown in (A). Scale bar = 50 μm. (B, C) Quantitative analysis of the protein intensities of FtH and FtL in the SCN and cortex regions from two separate fields for each mouse. The data are presented as the mean ± SEM, *n* = 3. (D) Hippocampal FtH and FtL proteins, as evaluated by western blot analysis. (E) Quantitative analysis of FtH and FtL protein levels from the western blot data, compared to the *Fpn1* ^flox/flox^ group, after normalizing to the respective β‐actin expression. The data are presented as the mean ± SEM, *n* = 3. The locomotor activities of the mice were measured using a Mouse Wheel Running Activity Monitoring System. The monitoring data represent the distance traveled for each animal per second (cm/s). (F, I) Representative double‐plotted actograms of spontaneous locomotor activity of *Fpn1* ^flox/flox^ and *Fpn1* ^*Cdh5*^‐cKO mice (F) or WT (control) and APP/PS1 mice (I) that were kept in a standard photoperiod of 12 h light (L) and 12 h dark (D) for 6 days, followed by 48 h of constant darkness. In these plots, black bars indicate activity and gray boxes indicate periods of darkness. (G) Average autonomous activities of the *Fpn1* ^flox/flox^ and *Fpn1* ^*Cdh5*^‐cKO mice from 12:00 a.m. to 12:00 p.m. (H) Total activity of the *Fpn1* ^flox/flox^ and *Fpn1* ^*Cdh5*^‐cKO mice per day during the experimental period. (J, K) Daily (J) and total (K) activities for the WT and APP/PS1 mice. (L, M) Serum melatonin levels in the indicated groups. All the data are presented as the mean ± SEM. The numbers of mice (*n*) for each group are indicated in (G, H), (J, K), and (L, M). Statistical analysis was performed using a two‐tailed Student's *t*‐test; *p < 0.05, **p < 0.01, ***p < 0.001, compared with the *Fpn1* ^flox/flox^ group or the WT group.

FPN1 is the only protein known to export cellular iron in mammals.^28^ Conditional knockout of FPN1 in cerebral microvascular endothelial cells (*Fpn1* ^*Cdh5*^‐cKO) inhibits the transport of peripheral iron into the brain through the blood–brain barrier (BBB), leading to brain iron deficiency.^20^ FtH and FtL are cellular iron storage proteins that are usually used as indicators of uncommitted iron levels.^20^ In the present study, we first examined the distribution and expression of FtH and FtL, in the SCN, cortex, and hippocampus of 10‐month‐old *Fpn1* ^flox/flox^ and *Fpn1* ^*Cdh5*^‐cKO mice. The *Fpn1* ^*Cdh5*^‐cKO mice exhibited lower FtH and FtL levels in these brain regions compared to the controls, which is consistent with decreased iron, as we previously described in these mice (Figure 1A–E).^20^

We next assessed the locomotor rhythms in the *Fpn1* ^*Cdh5*^‐cKO mice. The results show that *Fpn1* ^*Cdh5*^‐cKO mice had significantly increased locomotor activity compared to the *Fpn1* ^flox/flox^ mice (Figure 1F–H). Several studies have demonstrated atypical brain iron accumulation in aged APP/PS1 mice.^21^, ^29^ In contrast to the *Fpn1* ^*Cdh5*^‐cKO mice, the APP/PS1 transgenic mice showed inhibited rhythmic activities compared to the WT mice which may be related to the elevated brain iron levels in these mice (Figure 1I–K).

Melatonin plays a crucial role in mammalian circadian rhythms, exhibiting diverse secretion levels during the light/dark phases of a day. Additionally, this neurohormone serves as a potent antioxidant.^30^, ^31^ In our study, we conducted an ELISA assay using a mouse melatonin ELISA kit to measure serum melatonin levels. Our findings revealed a significant decrease in melatonin levels in *Fpn1* ^*cdh5*^‐CKO mice compared to the *Fpn1* ^flox/flox^ control group. Conversely, in APP/PS1 mice with brain iron deposition, we observed greater serum melatonin levels than in the WT group (Figure 1L,M). These results demonstrate contrasting changes in serum melatonin levels in these two transgenic mice groups, which aligns with the hormone's role in circadian rhythm regulation. Moreover, our study suggests a potential link between brain iron status and circulating melatonin levels. Thus far, our results imply that lower brain iron levels in aging mice may promote locomotor activity and interfere with circadian rhythms.

### 3.2 Brain iron deficiency leads to variation in the molecular circadian clock in *Fpn1* ^*Cdh5*^‐cKO mice

**FIGURE 2:**
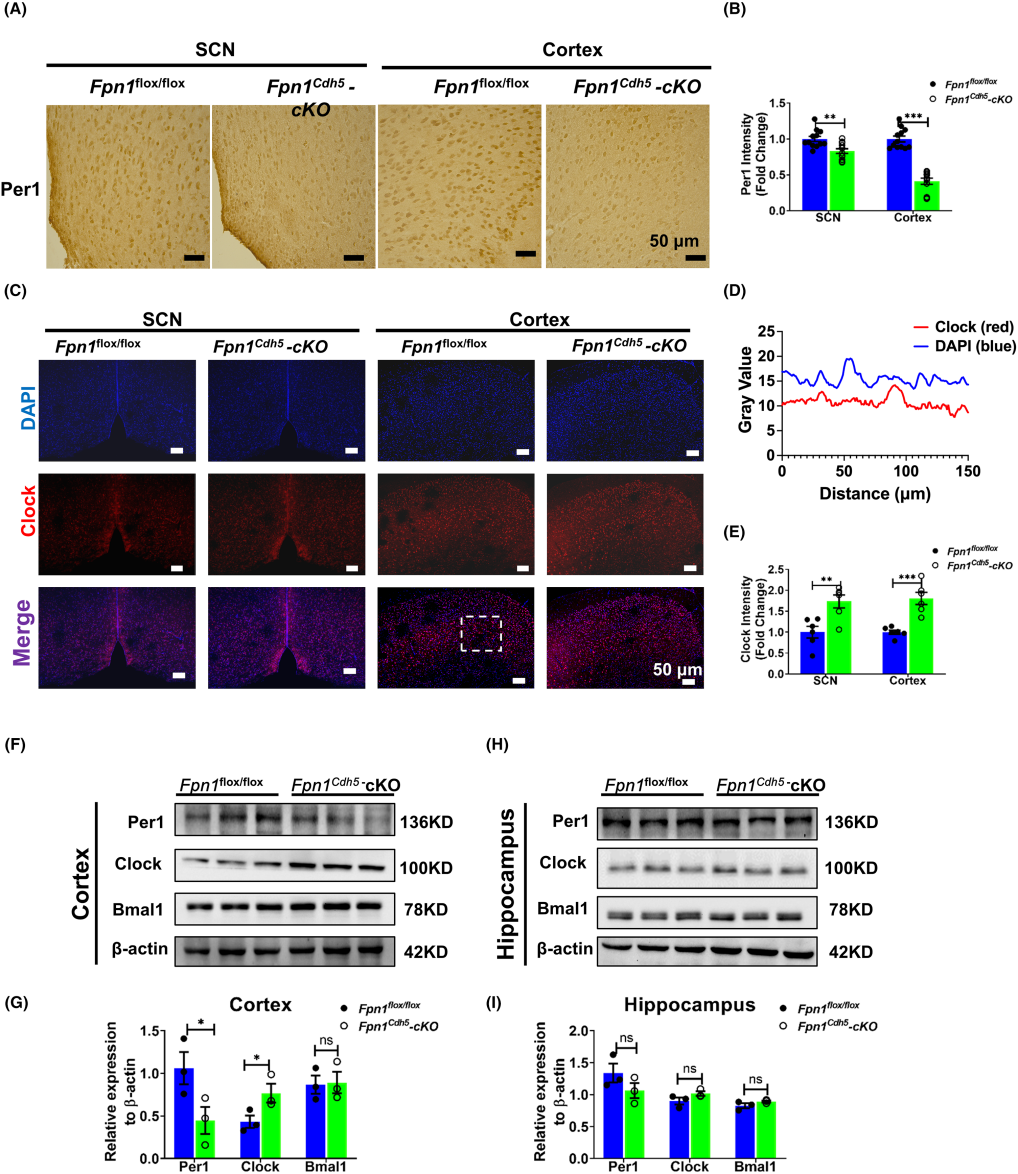
Distribution and expression of Per1 and Clock in the SCN, cortex, and hippocampus regions of *Fpn1* ^flox/flox^ and *Fpn1* ^*Cdh5*^‐cKO mice. (A) Per1 distribution in the SCN and cortex, as detected by IHC. (B) Average intensity of Per1 in these regions, for each mouse. The data were calculated from four separate fields. The data are presented as the mean ± SEM, *n* = 3. Scale bar = 50 μm. (C) Double‐immunofluorescence assays were performed to detect the distribution and expression of Clock in the SCN and cortex. Scale bar = 50 μm. (D) The intensity profile (dashed box in panel C of the signals from both fluorescent channels indicates the colocalization of Clock and DAPI). (E) Quantified average intensity of Clock in the SCN and Cortex regions from two separate fields for each mouse. The data are presented as the mean ± SEM, *n* = 3. (F–I) Representative western blots for the expression levels of Per1, Clock, and Bmal1 in the cortex (F) and hippocampus (H) and quantification of the western blot analysis results in the cortex (G) and hippocampus (I). The data are presented as the mean ± SEM, *n* = 3. Statistical analysis was performed using a two‐tailed Student's *t*‐test; *p < 0.05, **p < 0.01, ***p < 0.001, compared with the *Fpn1* ^flox/flox^ group, “ns” refers to no significance.

The circadian rhythm is produced and regulated by the so‐called “clock” genes. In the feedback loop composed by clock genes and their encoded proteins, the BMAL1/CLOCK complex acts as a transcriptional activator of the genes encoding the repressor proteins, PER1 and PER2.^32^, ^33^ In the present study, we examined the expression of PER1, CLOCK, and BMAL1 to investigate whether the decreased brain iron in *Fpn1* ^*Cdh5*^‐cKO mice could impact the levels of these proteins. Immunostaining results revealed that there was an apparent decrease in PER1 and increased expression of CLOCK in the SCN and cortex of *Fpn1* ^*Cdh5*^‐cKO mice (Figure 2A–E). Aligned with these results, western blot assays using cortical and hippocampal samples also revealed elevated CLOCK and diminished PER1 protein in cortex (Figure 2F,G). We did not see significant changes in PER1 and CLOCK expression in the hippocampus (Figure 2H,I). Collectively, our data suggest that alterations in brain iron levels can cause disturbances in circadian rhythms through the regulation of the TTFL composed by core clock genes.

### 3.3 Exogenous cellular iron can regulate the expression of PER1, BMAL1, and CLOCK in U251 cells

In subsequent experiments, we examined the molecular mechanisms of cellular iron regulation of clock genes in vitro using the human glioma cell line, U251. First, we assessed the endogenous, rhythmic expression of *PER1*, *BMAL1*, *CLOCK*, and cryptochrome 1 (*CRY1*) in U251 cells (Figure S1). We observed the anticipated, rhythmic transcription of these genes in vitro, which aligns with their rhythms in vivo conducted in *Fpn1* ^*Cdh5*^‐cKO mice. We then treated the U251 cells with different concentrations of the iron chelator, DFO, to deplete the cells of iron. Alternatively, we added ferric ammonium citrate (FAC) to the cells to generate an iron overload status. Based on cell viability, we selected 50 μM and 100 μM as the final concentrations of DFO and FAC, respectively (Figure S2).

**FIGURE 3:**
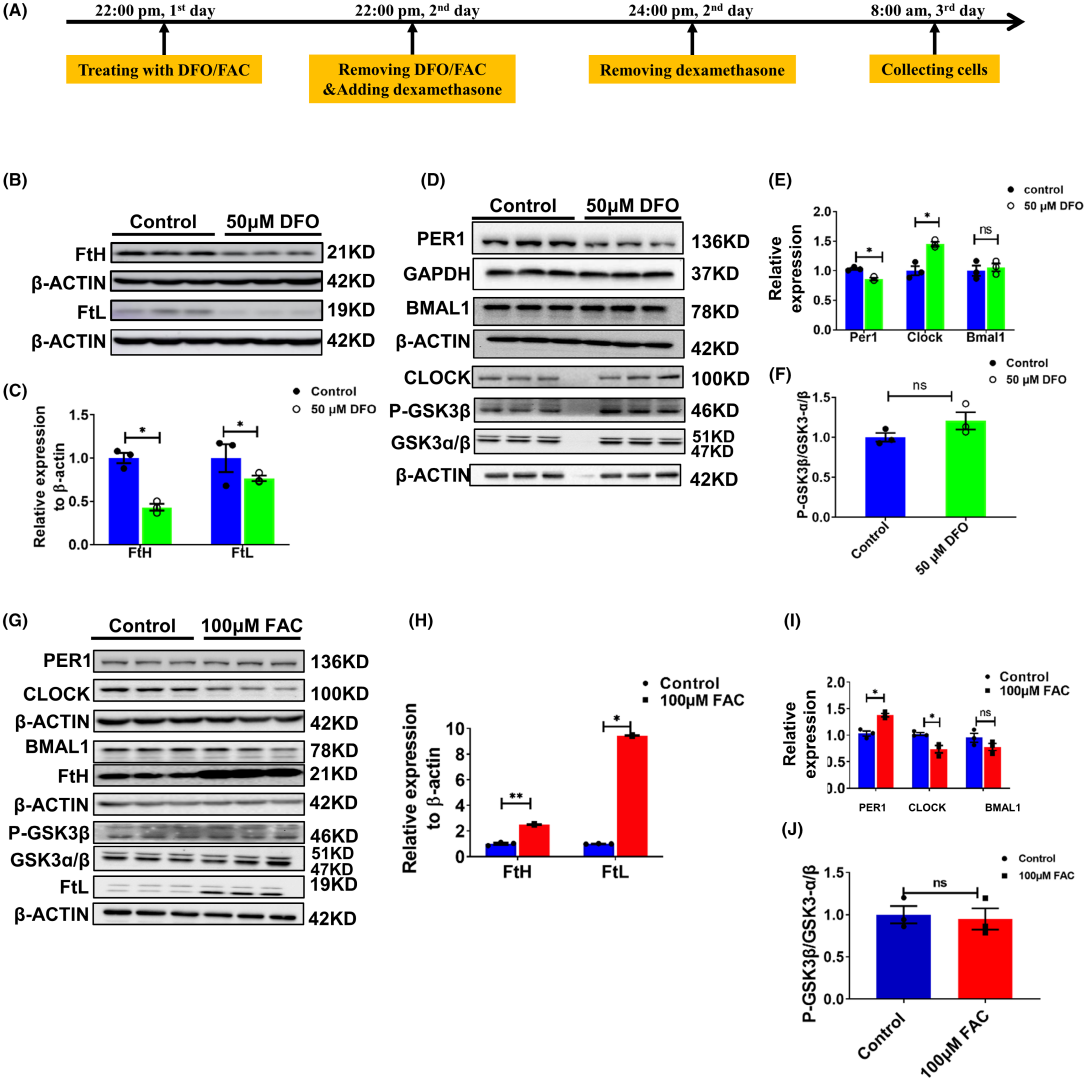
Altered expression of PER1, CLOCK, BMAL1 and P‐GSK3β in U251 cells treated with 50 μM DFO or 100 μM FAC at 8 am. (A) Schematic illustration of the treatment of cells with DFO or FAC; the cells were collected and detected at 8 a.m. (B) Representative western blot analysis results for FtH and FtL in U251 cells treated with 50 μM DFO. (C) Statistical analysis of the results from the experiment presented in panel B. FtH and FtL were compared to the control group after normalizing to the respective β‐ACTIN expression. (D) Protein levels of PER1, CLOCK, BMAL1 and p‐GSK3β, as detected by western blot analysis. (E) Statistical analysis of the results of the experiment shown in panel D. (F) Statistical analysis of phosphorylated GSK3β levels. The data represented in panels E and F were quantified after normalizing to the respective GAPDH or β‐ACTIN expression. (G) Representative western blot analysis results for PER1, CLOCK, BMAL1 FtH, p‐GSK3β, GSK3α/β and FtL, as detected by western blot analysis, in U251 cells treated with 100 μM FAC for 24 h. (H) Statistical analysis of FtH and FtL compared to the control group after normalizing to the respective β‐ACTIN expression. (I) Quantification of the results of PER1, CLOCK, and BMAL1 protein expression. (J) Statistical analysis of P‐GSK3β levels. The data represented in panels I and J were quantified after normalizing to β‐ACTIN expression. The data are presented as the mean ± SEM, *n* = 3. Statistical analysis was performed using a two‐tailed Student's *t*‐test; *p < 0.05, **p < 0.01 compared to the Control group. “ns” refers to no significance.

After subjecting U251 cells to a 24 h treatment with 50 μM DFO, we selected two specific time points, 8:00 a.m. and 8:00 p.m., to explore the impact of cellular iron alterations on the protein expression of core clock genes. Figure 3 presents the results obtained from U251 cells collected at 8:00 a.m., while Figure S3 shows the protein levels of clock genes in U251 cells collected at 8:00 p.m.

In Figure 3, we first examined the protein levels of PER1, CLOCK, and BMAL1. Since BMAL1 can also be down‐regulated by the p‐GSK3β/GSK3β pathway,^34^, ^35^ we evaluated the phosphorylation of GSK3β. Our results show diminished levels of FtH and FtL protein, indicating an iron starvation status in the DFO‐treated cells. Additionally, PER1 protein levels were significantly decreased, while CLOCK levels exhibited an apparent increase, which is consistent with its down‐regulation via PER1.^33^ The western blot results also show markedly greater levels of phosphorylated GSK3β; however, we did not observe significant alterations in BMAL1 expression (Figure 3A–F). The latter is probably because of the bi‐directional regulation of BMAL1 by PER1 and the p‐GSK3β/GSK3β pathway. Our results preliminarily demonstrate that cellular iron status influences the expression of the circadian clock genes.

To examine whether iron supplementation can shift clock gene expression in the opposite direction of that observed upon chelation, we treated the U251 cells with FAC. As expected, the protein level of PER1 was increased, while CLOCK protein was diminished compared to the control cells. We did not see significant changes in the expression of BMAL1 and phosphorylated GSK3β (Figure 3G–J).

In order to validate the results obtained from U251 cells, we utilized the MA‐c cell line and performed a qRT‐PCR assay to examine the mRNA expression pattern of clock genes, including *Bmal1*, *Clock*, *Per1*, and *Cry1*. The mRNA results demonstrate that the expression of clock genes in mouse MA‐c cells maintained circadian rhythmicity, which is in contrast to the mRNA results obtained from human U251 cells (Figure S4). Additionally, we investigated the impact of altered cellular iron levels on the protein expression of Per1, at 8:00 a.m. and 10:00 p.m., in MA‐c cells (Figures S5 and S6). The findings in MA‐c cells reveal that the addition of FAC enhanced PER1 expression, while DFO inhibited it. Collectively, these results in MA‐c cells further support a potential molecular connection between cellular iron and PER1 expression. Taken together, our results show that altered iron levels influence the expression of the core clock genes, including PER1, CLOCK, and BMAL1, in vitro.

### 3.4 Endogenous overexpression of FPN1 and TfR1 induces changes in cellular iron levels in U251 cells

**FIGURE 4:**
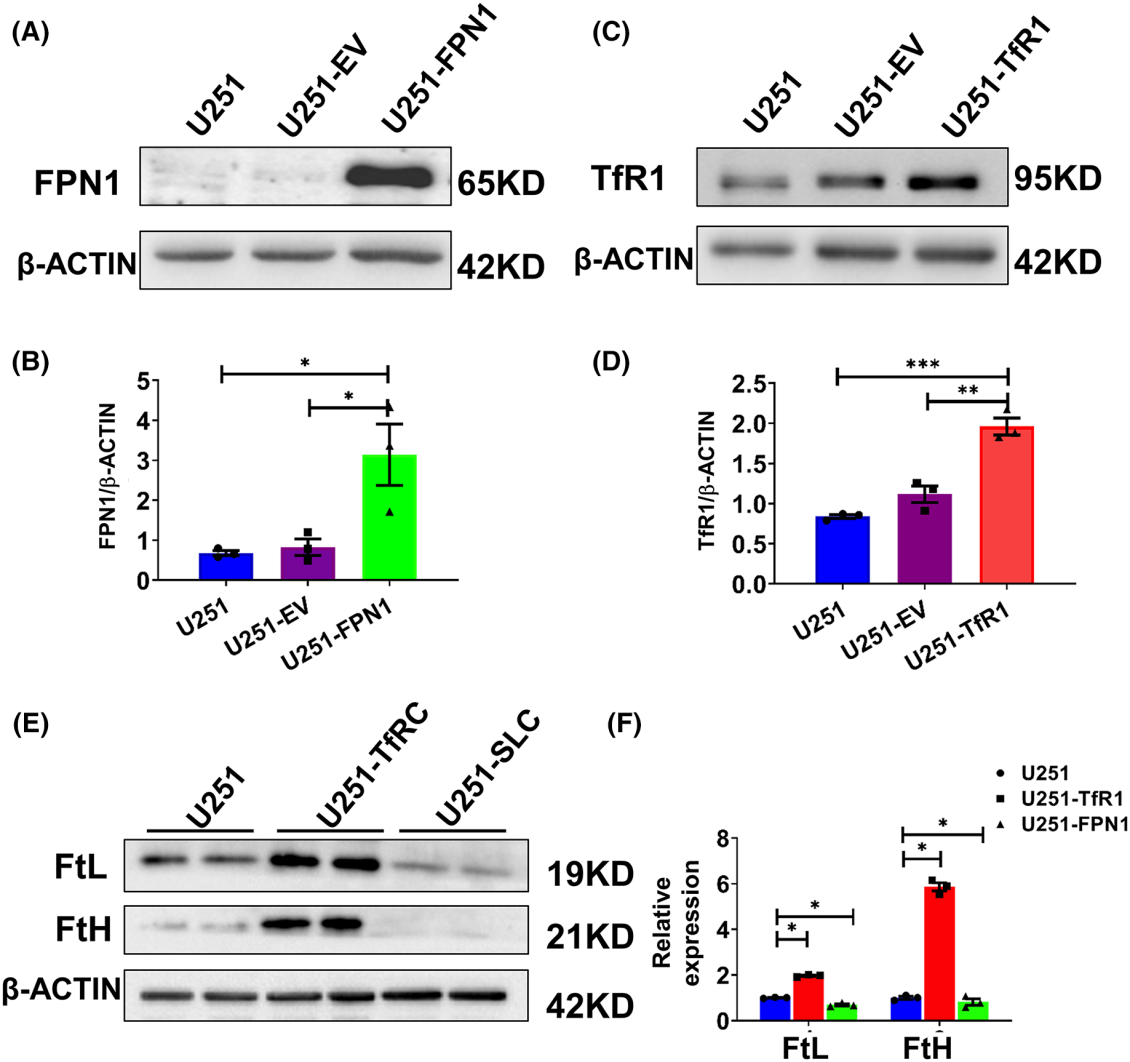
Construction and verification of U251 cells endogenously overexpressing FPN1 and TfR1. After generating U251 cells stably overexpressing FPN1 or TfR1, we evaluated the protein levels of FPN1 and TfR1 by western blot analysis. (A, C) Western blot analysis results for FPN1 and TfR1 in U251, U251 cells transfected with empty vector (U251‐EV), and U251 cells overexpressing FPN1 (U251‐FPN1) or TfR1 (U251‐TfR1). (B, D) Quantification of the results of the experiments presented in panels A and C. The protein levels were normalized to β‐ACTIN expression. The data are presented as the mean ± SEM, *n* = 3. Statistical analysis was performed using a two‐tailed Student's *t*‐test; *p < 0.05, **p < 0.01, ***p < 0.001. (E) Western blot analysis results for FtL and FtH expression in U251‐TfR1 and U251‐FPN1 cells. (F) Statistical analysis for FtH and FtL compared to the U251 group after normalizing to the respective β‐ACTIN expression. The data are presented as the mean ± SEM, *n* = 3. Statistical analysis was performed using a two‐tailed Student's *t*‐test; *p < 0.05.

To support our hypothesis that altered cellular iron levels influence the expression of clock genes, we constructed two cell models that endogenously overexpress FPN1 (U251‐FPN1) or TfR1 (U251‐TfR1). In the process of cellular iron transport, TfR1 is the gateway for import of iron into cells. As expected, overexpression of TfR1 induced enhanced iron import and subsequent iron accumulation in U251 cells, while the U251‐FPN1 cells exhibited lower cellular iron content, as evidenced by increased FtH and FtL expression in the U251‐TfR1 cells and decreased protein levels of FtH and FtL in the U251‐FPN1 cells (Figure 4).

### 3.5 The levels of PER1, CLOCK, and BMAL1 proteins are altered in U251‐FPN1 and U251‐TfR1 cells

**FIGURE 5:**
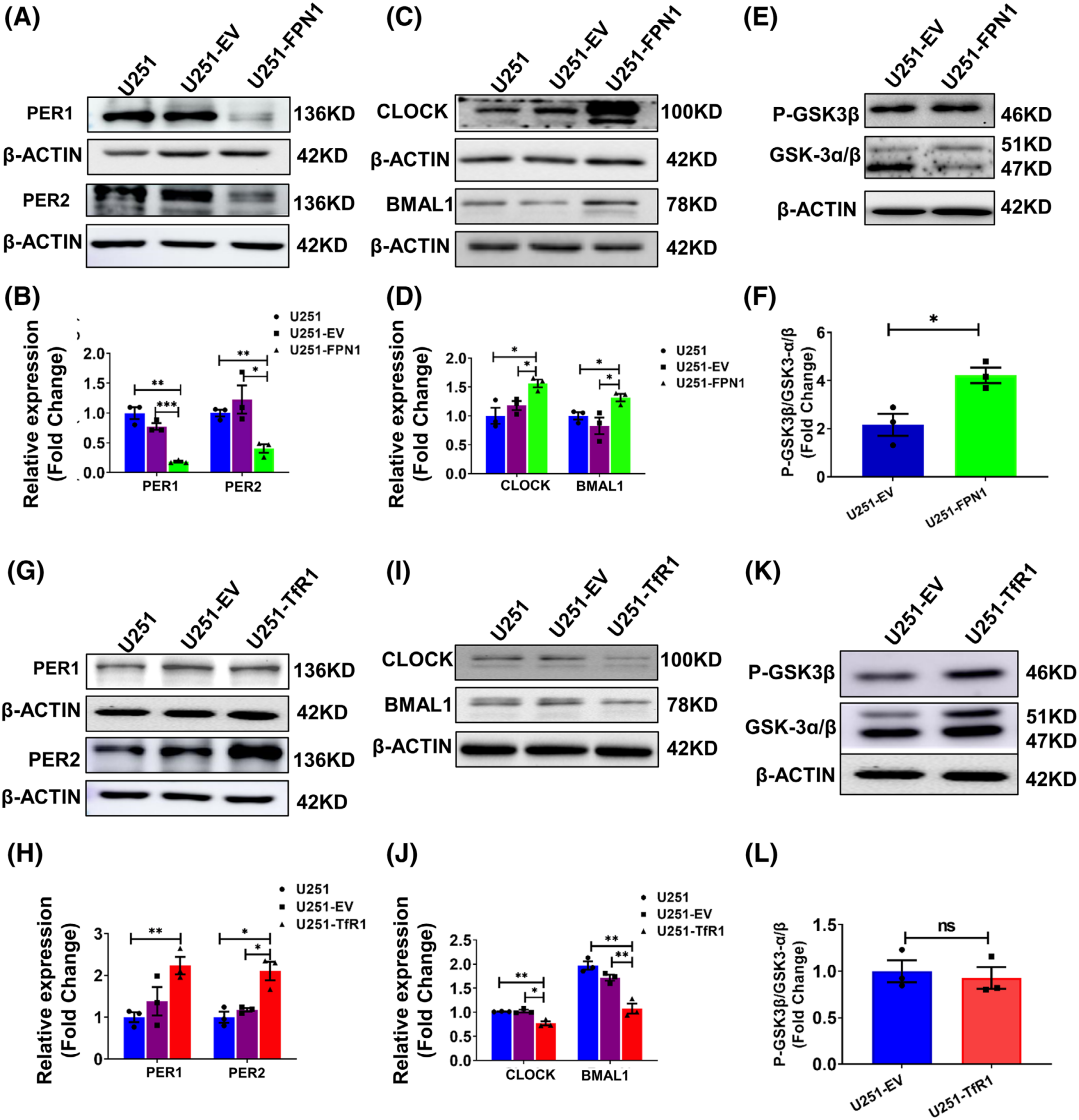
Protein levels of PER1, PER2, CLOCK, BMAL1, and p‐GSK3β in U251‐FPN1 and U251‐TfR1 cells. (A) Western blot analysis results for PER1, PER2 in U251 cells, U251 cells with empty vector (U251‐EV), and U251 cells overexpressing FPN1 (U251‐FPN1). (C) Representative western blot analysis of CLOCK and BMAL1 in these three groups. (E) Levels of phosphorylated GSK3β and GSK‐3α/β in the U251‐EV and U251‐FPN1 groups. (B, D, F) Statistical analysis of the expression of the indicated proteins compared to the U251 or U251‐EV group after normalizing to the respective β‐ACTIN expression. The data are presented as the mean ± SEM, *n* = 3. (G, I) Representative western blot analysis results for PER1, PER2, BMAL1, and CLOCK in U251, U251‐EV, and U251 cells overexpressing TfR1 (U251‐TfR1). (K) Levels of phosphorylated GSK3β in the indicated groups. (H, J, L) Statistical analysis for the indicated proteins compared to the U251 or U251‐EV group after normalizing to the respective β‐ACTIN expression. The data are presented as the mean ± SEM, *n* = 3. Statistical analysis was performed using a two‐tailed Student's *t*‐test; *p < 0.05, **p < 0.01, “ns” refers to no significance.

Next, we assessed the levels of PER1, PER2, BMAL1, and CLOCK in the U251‐FPN1 and U251‐TfR1 cells. In the U251‐FPN1 cells, we observed considerably reduced expression of PER1 and PER2, while, accordingly, the levels of CLOCK and BMAL1 were increased (Figure 5A–D). This effect is likely to have occurred via elevated levels of p‐GSK3β in the cells (Figure 5E,F). These results align with those we observed following iron chelation. In the U251‐TfR1 cells, with their elevated cellular iron status, the levels of PER1 and PER2 were increased, while the CLOCK and BMAL1 proteins were decreased. However, we did not observe measurable alterations in p‐GSK3β levels (Figure 5G–L). These results from U251‐TfR1 cells are consistent with the results in U251 cells treated with FAC. Our results further strengthen the hypothesis that the expression of core clock genes can be regulated by altered cellular iron levels.

### 3.6 The mechanism of iron regulation of circadian rhythms may be via changes in PER1

**FIGURE 6:**
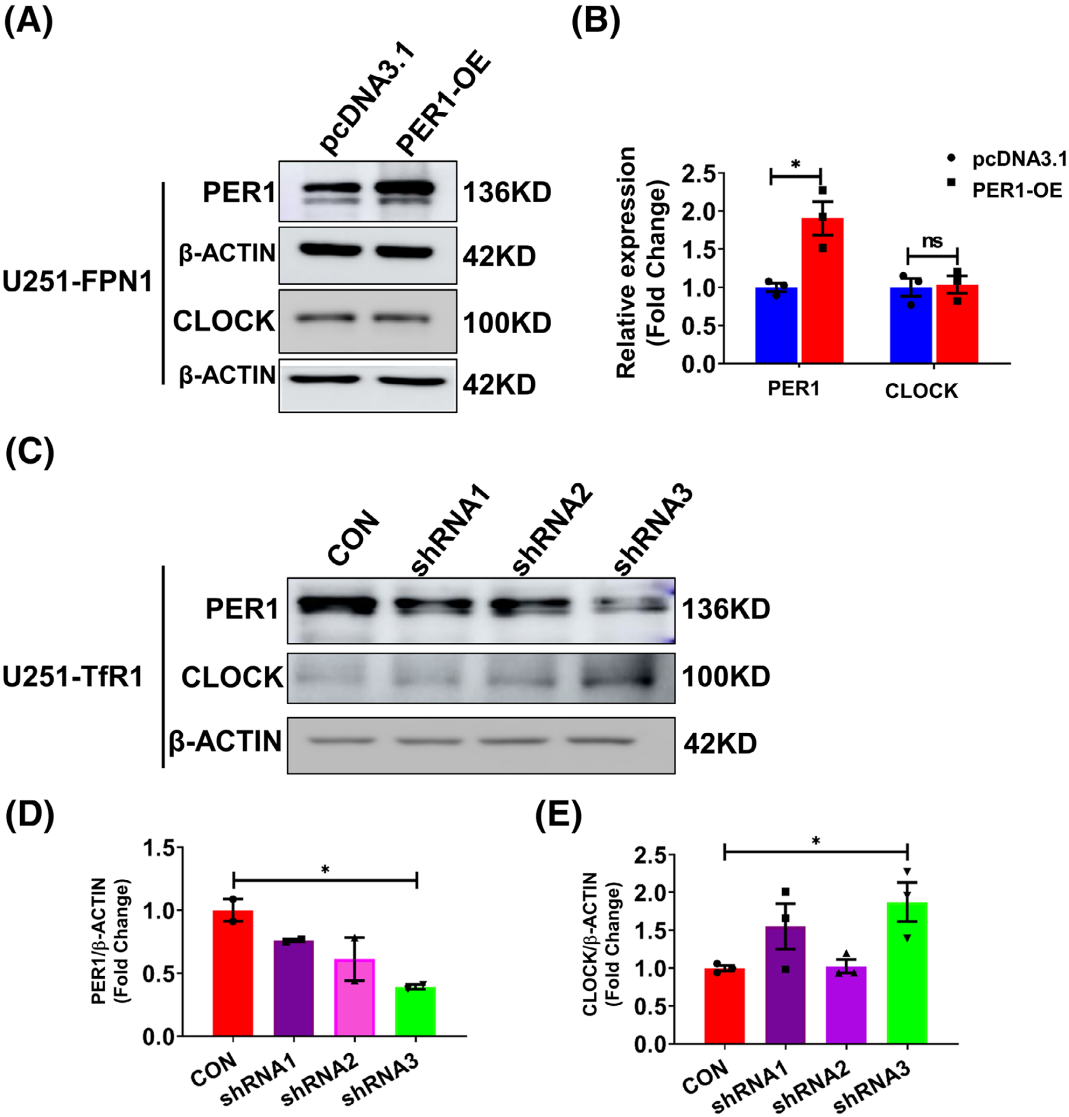
Expression levels of CLOCK protein in U251‐FPN1 and U251‐TfR1 cells with modified Per1 expression. (A) Representative western blot analysis results of PER1 and CLOCK in U251‐FPN1 cells overexpressing PER1. (B) Quantification of the experiment presented in panel A. The data are presented as the mean ± SEM after normalizing to the respective β‐ACTIN expression, *n* = 3. (C) Western blot analysis results of PER1 and CLOCK proteins in U251‐TfR1 cells with inhibited expression of PER1 using *Per1*‐shRNAs. (D, E) Quantified results of PER1 and CLOCK expression from the experiment shown in panel C. The data are presented as the mean ± SEM after normalizing to the respective β‐ACTIN expression. *n* = 3, *p < 0.05, “ns” refers to no significance.

The above results indicate that decreased cellular iron can cause a decline in the levels of PER1, which in turn leads to elevated expression of CLOCK, which is down‐regulated by PER1. Therefore, we conjectured that PER1 is directly influenced by cellular iron levels. To test this, we increased PER1 levels in U251‐FPN1 cells through transfection with a plasmid‐overexpressing PER1 (*Per1*‐OE). In U251‐FPN1 cells with PER1 overexpression, we detected a decreased trend in CLOCK protein levels, although the difference was not statistically significant (Figure 6A,B). On the other hand, we also decreased PER1 levels in U251‐TfR1 cells via *Per1*‐shRNAs. After shRNA knockdown of *Per1*, CLOCK protein levels were significantly increased compared to the controls (Figure 6C–E), suggesting that the mechanism of iron's effect on the TTFL may be through changes in PER1 protein levels.

## 4 CONCLUSION

Aging is characterized by progressive impairments in physiological functions in the body, including the robustness of the circadian clock, that can lead to disturbed sleep–wake cycles and lowered synchronization of circadian rhythms, which in turn accelerates the process of aging.^33^, ^36^ Numerous studies have examined the pathogenesis of aging‐related circadian rhythm disruption, including disturbances in the autonomic nervous system, hormone levels, or immune system.^37^, ^38^, ^39^ However, the underlying cause(s) of the changes to the circadian rhythm with age largely remain enigmatic. As one of the most abundant trace elements in the brain, iron contributes to various essential physiological processes, including oxygen transport, DNA synthesis, and oxidative phosphorylation.^10^ Numerous studies have shown that progressive iron accumulation is a major risk factor in aging and is connected to neurodegenerative diseases, such as AD and PD.^29^, ^40^, ^41^ Aberrant brain iron increases with age, resulting in dysfunction of the central nervous system, since iron‐induced redox stress can accelerate cellular senescence and neurodegeneration. It has also been found that excessive iron accumulation is usually accompanied by sleep–wake phase disorders, which are closely related to the circadian rhythm.^42^, ^43^ Yet, the apparent relationship between elevated brain iron levels and circadian clock desynchrony in the elderly, and the mechanisms underlying these effects, represent a gap in our knowledge.

In the present study, we explored whether altered brain iron levels can lead to changes in behavioral rhythm, and the mechanisms involved. In our study, we used *Fpn1* ^*Cdh5*^‐cKO mice as a model of brain iron deficiency since conditional knock of *Fpn1* in microvascular endothelial cells blocks iron import into the brain through the BBB.^20^ In addition, previous studies have reported elevated iron levels in the cortex and hippocampus of APP/PS1 mice.^21^, ^29^ In our present study, *Fpn1* ^*Cdh5*^‐cKO mice exhibited enhanced activity rhythms, while APP/PS1 inhibited activities. It is worth noting that the observed changes in locomotor activity rhythms in the APP/PS1 mice with brain iron overload are consistent with a previous study.^44^ In this study, we assessed the levels of the cellular iron storage proteins, namely FtH and FtL, in various brain regions, including the SCN, cortex, and hippocampus, of the *Fpn1* ^*Cdh5*^‐cKO mice. We found that these proteins were decreased compared to age‐matched controls, which is consistent with lower uncommitted iron levels in these brain regions. The increased FtH and FtL levels have already been reported in our previous study. Since both of these mouse models exhibited disturbances in locomotor rhythm (Figure 1), we conclude that there is a connection between iron levels and the regulation of circadian rhythms.

There has been robust study on melatonin and circadian rhythms. Melatonin is not only examined with respect to its effects on circadian rhythms but also various other functions, such as antioxidant effects.^30^, ^31^ A series of studies have demonstrated that melatonin can alleviate neural damage and cell death caused by excessive iron.^45^, ^46^ In the present study, we observed a significant increase in serum melatonin levels in APP/PS1 mice, while *Fpn1* ^*cdh5*‐^cKO mice with brain iron deficiency showed the opposite trend. This result suggests a potential correlation between brain iron levels and melatonin levels. More importantly, it implies that elevated brain iron in APP/PS1 mice may lead to increased oxidative stress levels, triggering a negative feedback mechanism to release melatonin. This hypothesis still requires further experimental validation, but it is consistent with our findings that brain iron has a significant role in regulating circadian rhythms.

In order to further understand the effects of brain iron on the shifts in the circadian rhythm, we evaluated the expression of core clock genes, including PER1, CLOCK, and BMAL1 in the SCN, cortex, and hippocampus in 10‐month‐old *Fpn1* ^*Cdh5*^‐cKO mice. We observed a diminished expression of PER1 and increased CLOCK expression in the SCN and cortex regions compared to controls, suggesting that brain iron status can have an influence on the expression of clock genes. Several previous studies revealed that peripheral iron accumulation plays a key role in aging‐related shifts of circadian function; sleep disturbances have been frequently seen in iron overload‐related diseases.^47^, ^48^ On the other hand, iron supplementation in young mice (4‐week‐old) yields indistinguishable or altered expression of *Per1* and *Per2* in the liver.^18^ Some additional studies have aimed to connect iron metabolism and circadian rhythms. The autophagy‐mediated degradation of BMAL1 favors lipid peroxidation and cell death in human tumor cell lines.^49^ Dietary iron may affect circadian glucose production through heme synthesis, which has also been demonstrated to modulate liver PER1 and PER2 expression in mice.^50^, ^51^ Heme can also function as a cofactor of clock genes and thus regulate biological cycles.^52^, ^53^ In addition, iron accumulation can also affect the expression of core clock genes, including PER1 and PER2, through histone methylation.^18^ In the present study, we found that changes in brain iron status can influence the expression of PER1, CLOCK, and BMAL1 in various brain regions of 10‐month‐old mice. Together, our results suggest that iron accumulation may drive disturbances in circadian and locomotor activity rhythms.

Astrocytes can act as active participants in the SCN network and display robust rhythms and TTFL function. Moreover, the astrocytic TTFL clock in the SCN is sufficient to determine behavioral rhythms.^57^ In contrast, disruption of the astrocytic TTFL lengthens behavioral and ex vivo SCN periods.^55^, ^56^ Using the U251 glioma cell model, we also observed rhythmic transcriptions of the clock genes, including *BMAL1*, *CLOCK*, *CRY1*, and *PER1*, which adds novel evidence that the biological clock also functions in vitro,^54^ suggesting that this cell line is suitable for biological rhythm research.

In the present study, we treated U251 cells with the iron chelator DFO to deplete the cells of iron and mimic the in vivo iron‐starvation state. Furthermore, we constructed FPN1‐overexpressing U251 cells (U251‐FPN1) to generate an iron‐depleted phenotype. We then assessed the protein levels of PER1, CLOCK, and BMAL1, since in mammalian cells, the BMAL1‐CLOCKk heterodimer can drive the transcription of *Per* and *Cry1*. The PER‐CRY1 protein complexes then translocate into the nucleus and suppress CLOCK‐BMAL1 activity. Additionally, we also investigated the phosphorylation of GSK3β, as p‐GSK3β pathway has been reported to down‐regulate Bmal1 expression.^5^, ^34^, ^35^ We found up‐regulated protein levels of p‐GSK3β and decreased PER1 levels in the two iron‐deficient cell models. In contrast, p‐GSK3β and Per1 showed an opposite expression pattern in both FAC‐treated U251 cells and U251‐TfR1 cells that overexpress TfR1 and take up erroneously elevated amounts of iron.

The mRNA expression of the core clock genes, including PER1, CLOCK, BMAL1, and CRY1, in U251 cells exhibited rhythmicity, as indicated in Figure S1. Therefore, it is reasonable to suspect that the influence of altered cellular iron on the expression of these genes will be affected by the time point of cell collection. However, our findings demonstrate that the protein expression of PER1, CLOCK, and BMAL1 detected at 8:00 p.m. (Figure S3) is similar to that observed at 8:00 a.m. The data presented in Figure 3 from U251 cells suggest that cellular iron accumulation induced by FAC promotes the expression of PER1, while iron starvation caused by DFO chelation inhibits PER1 expression. It is noteworthy that the impact of iron on the expression of clock genes remains consistent, regardless of the time at which the cells are collected and subsequently measured. In our study, we also observed rhythmic mRNA expression of *Per1*, *Clock*, *Bmal1*, and *Cry1* in a mouse cell line, MA‐c. Moreover, the effects of cellular iron levels on the expression of Per1 are consistent across different time points of cell collection and subsequent measurement in MA‐c cells.

Considering all of these above results, we conjecture that PER1 plays a vital role as a key clock gene in the process of iron regulation of the biological rhythm. To further investigate this hypothesis, we examined the expression of CLOCK protein levels in U251‐FPN1 cells overexpressing PER1. Under these conditions, CLOCK protein levels were decreased. In contrast, knockdown of *Per1* using shRNA in U251‐TfR1 cells significantly increased the levels of CLOCK protein. These findings suggest that PER potentially acts as a molecular mediator of the shifted circadian rhythm induced by altered iron levels. Taken together, our in vitro data from human and mouse cell lines indicate that elevated cellular iron levels may disrupt the cellular circadian rhythm by positively regulating PER1 expression. These in vitro findings further reinforce and validate the conclusions drawn from the mouse brain experiments with altered brain iron status, as presented in Figures 1 and 2.

**FIGURE 7:**
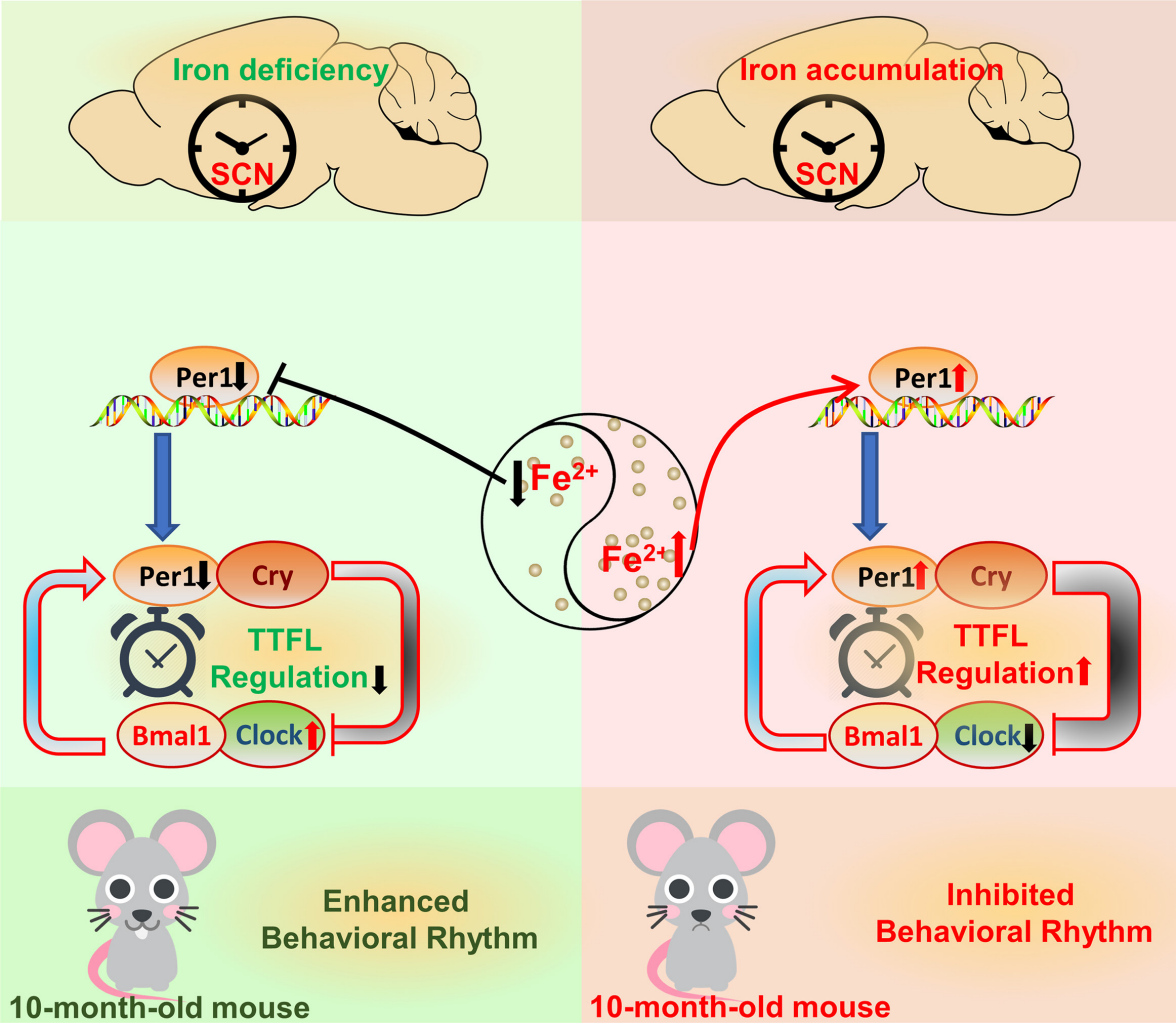
Schematic representation of the proposed molecular mechanism of diverse brain iron levels in circadian clock regulation. Decreased brain iron levels can inhibit the expression of Per1, which further inhibits TTFL regulation, resulting in elevated Clock protein and an enhancement of the behavioral rhythm. On the contrary, accumulated iron can induce increased Per1 level, therefore strengthening TTFL regulation, finally inhibiting rhythmic activity.

In summary, our study demonstrates that altered brain iron levels can affect the expression of clock genes in the brain, ultimately resulting in abnormal biological rhythm. Decreased cellular iron levels can inhibit the expression of PER1, which further inhibits TTFL regulation, resulting in elevated CLOCK protein and an enhancement of the behavioral rhythm. In contrast, iron accumulation induced increased PER1, strengthening TTFL regulation and therefore inhibiting rhythmic activity (Figure 7). PER1 may be a crucial clock gene mediating the shifted circadian rhythm during this process, however, mechanistic details require further investigation. Our study helps to close the gap in our understanding of age‐related dysregulation of the circadian rhythm and identifies brain iron regulation as a novel target for combating age‐associated disturbances of circadian rhythm.

## CONFLICT OF INTEREST STATEMENT

The authors declare that they have no conflicts of interest.

## Supporting information


Figure S1.

Figure S2.

Figure S3.

Figure S4.

Figure S5.

Figure S6.

Data S1.

Data S2.

## Data Availability

Data from this study are available from the corresponding author, Yan‐Zhong Chang and Peng Yu, upon reasonable request.
