# Supplementary figures and images for: Cellular iron depletion enhances behavioral rhythm by limiting brain Per1 expression in mice
Source: CNS Neurosci Ther. 2024 Feb 22;30(2):e14592. doi: 10.1111/cns.14592 (PMC10883092; doi:10.1111/cns.14592)

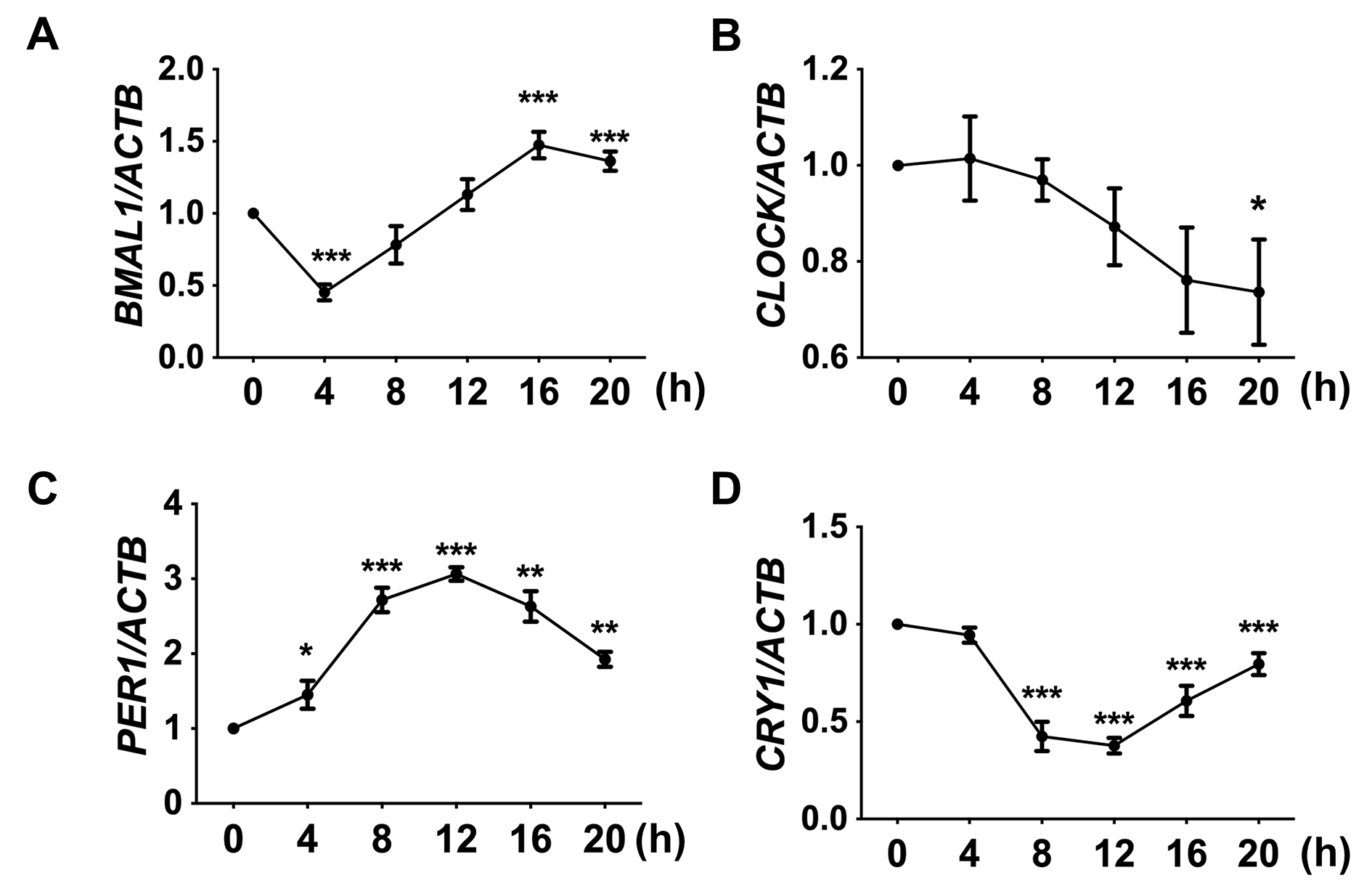

Supplement: Supplementary file 1 — Figure S1. [file CNS-30-e14592-s003.tif]

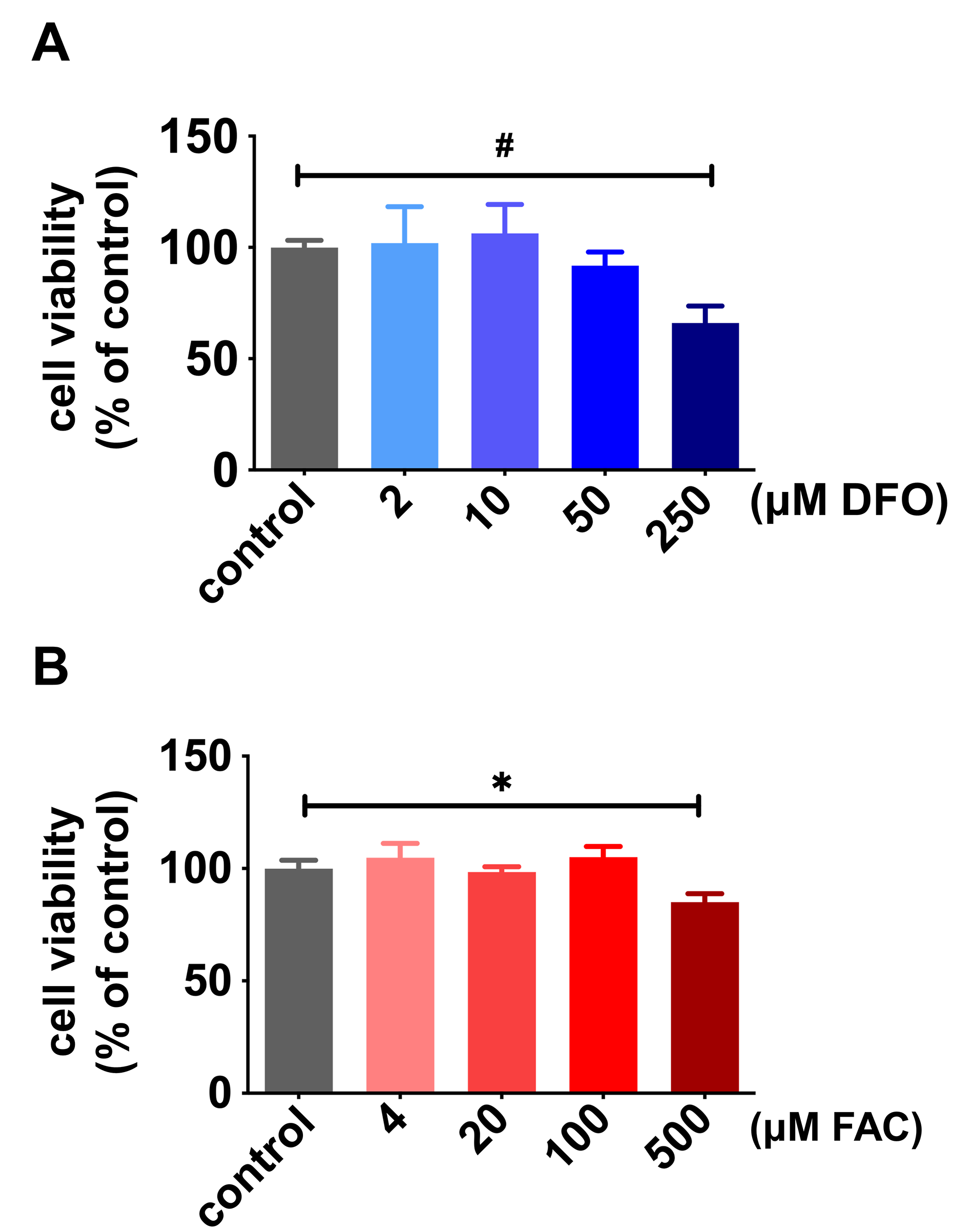

Supplement: Supplementary file 2 — Figure S2. [file CNS-30-e14592-s002.tif]

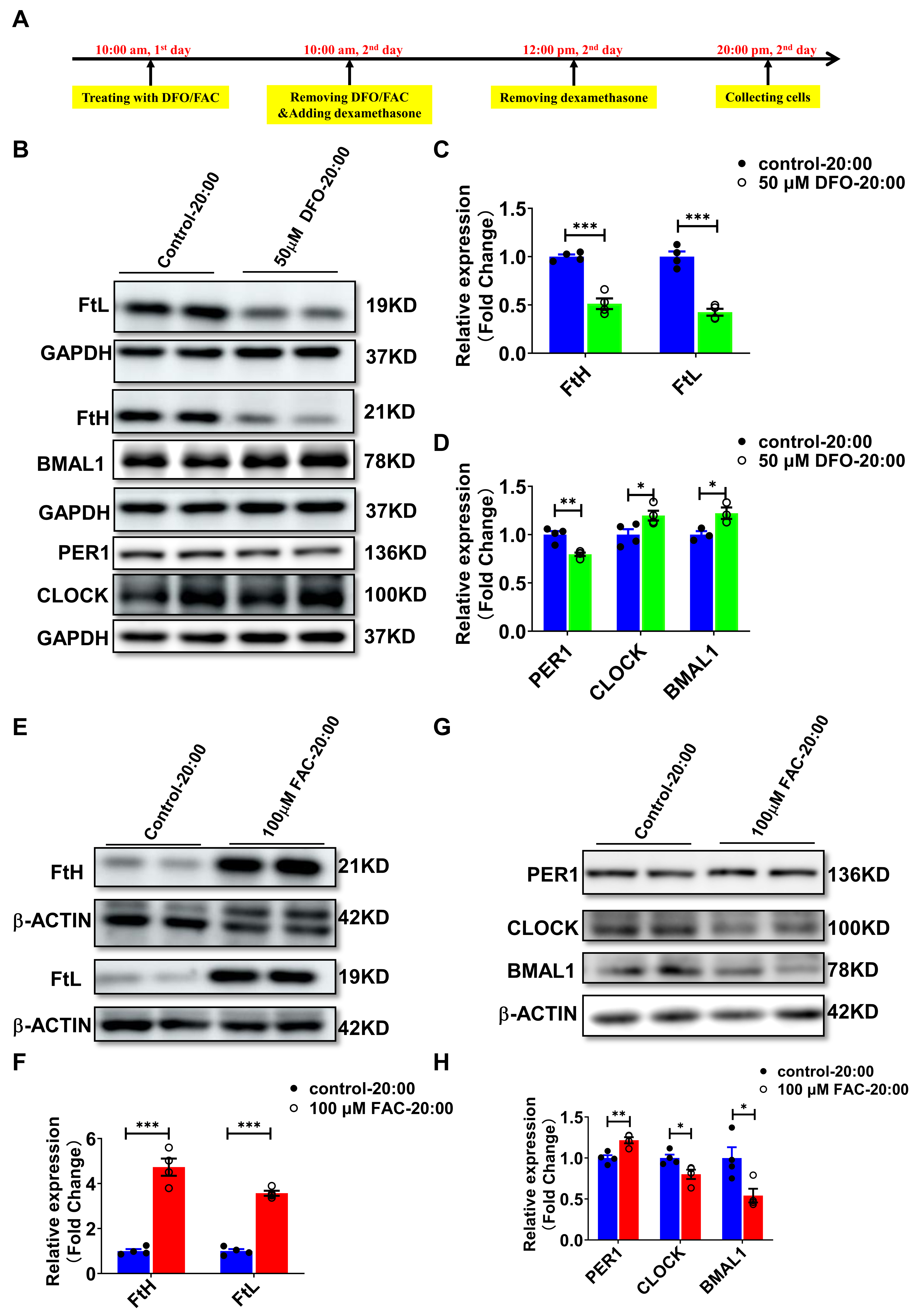

Supplement: Supplementary file 3 — Figure S3. [file CNS-30-e14592-s004.tif]

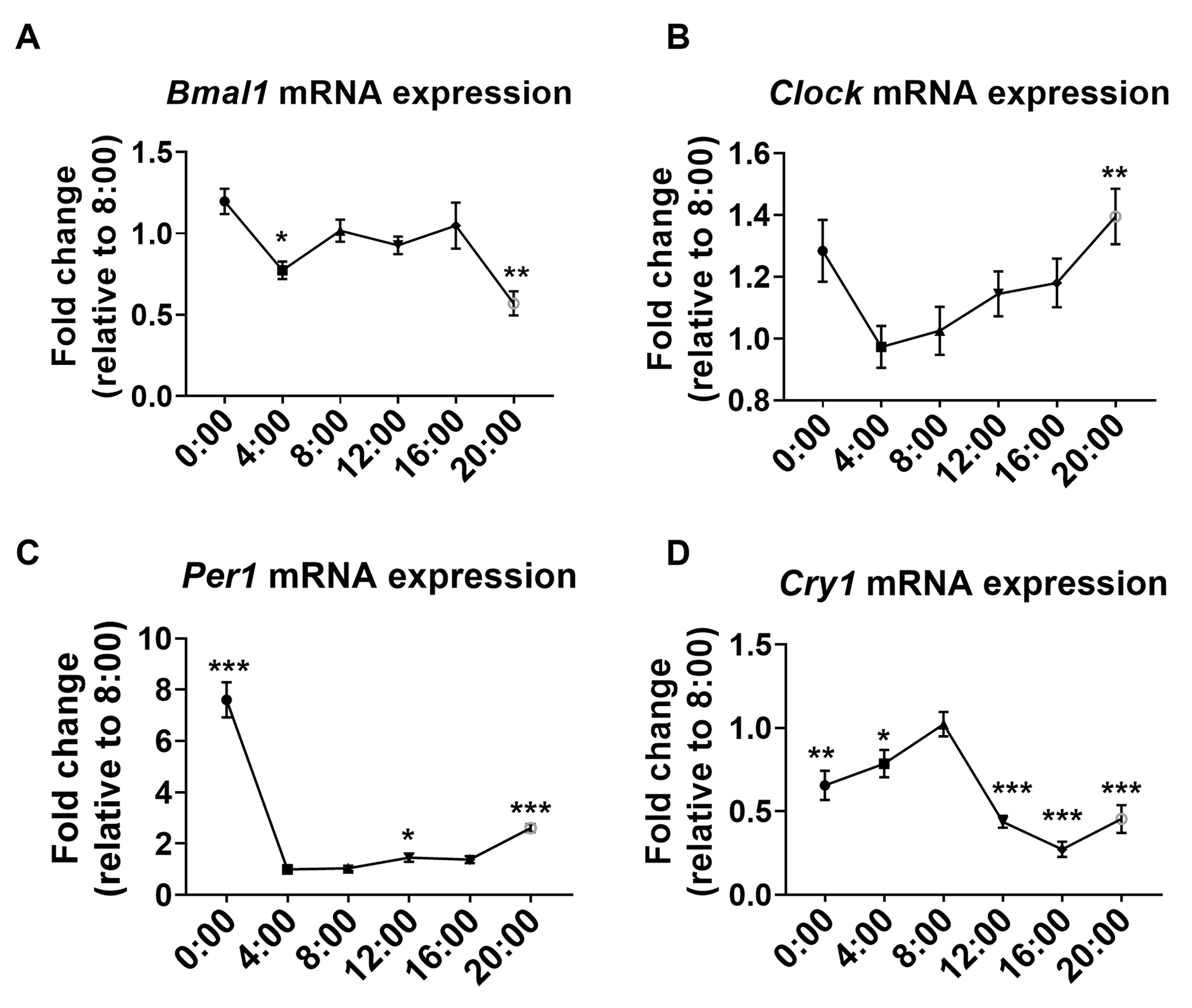

Supplement: Supplementary file 4 — Figure S4. [file CNS-30-e14592-s001.tif]

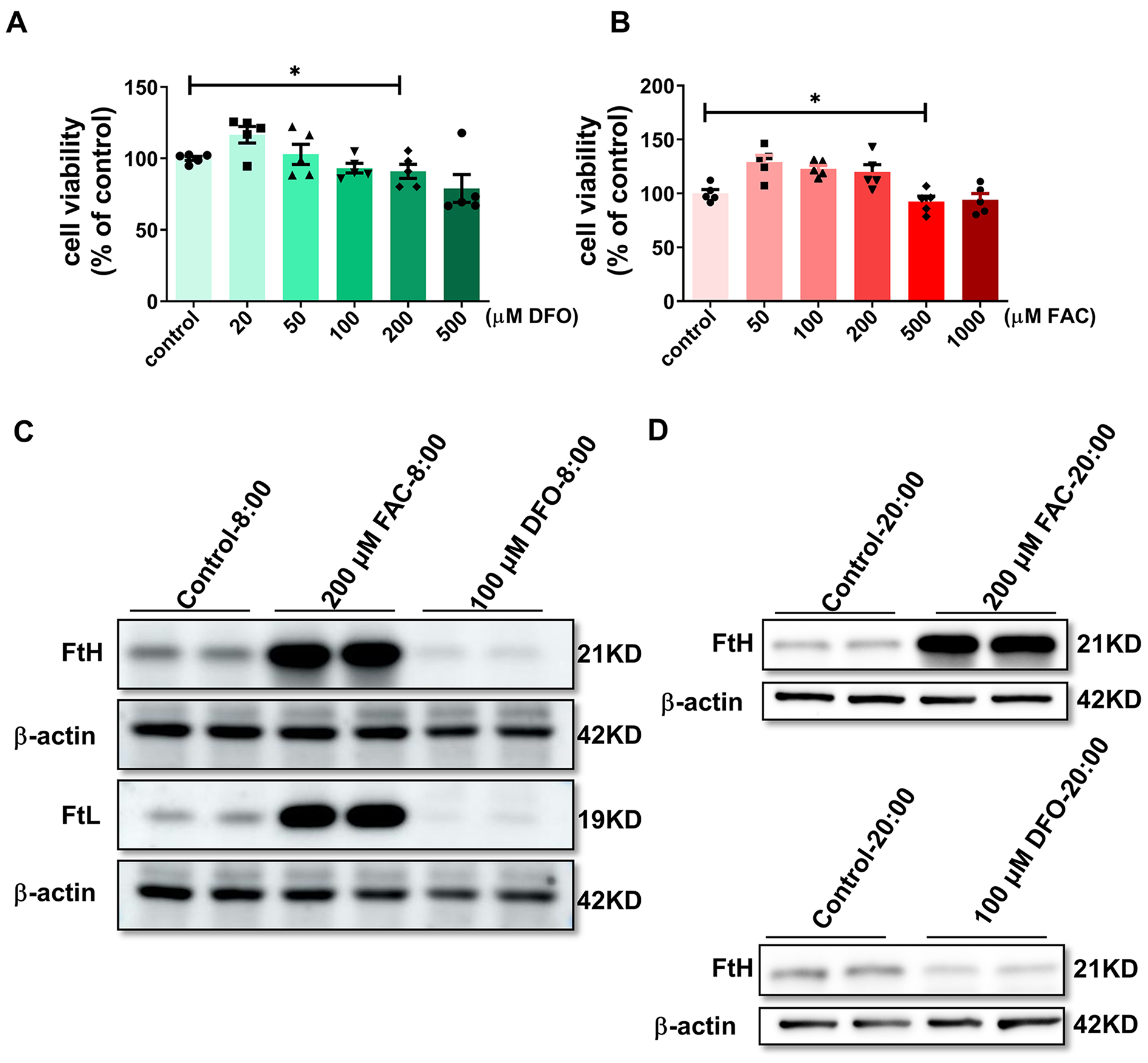

Supplement: Supplementary file 5 — Figure S5. [file CNS-30-e14592-s008.tif]

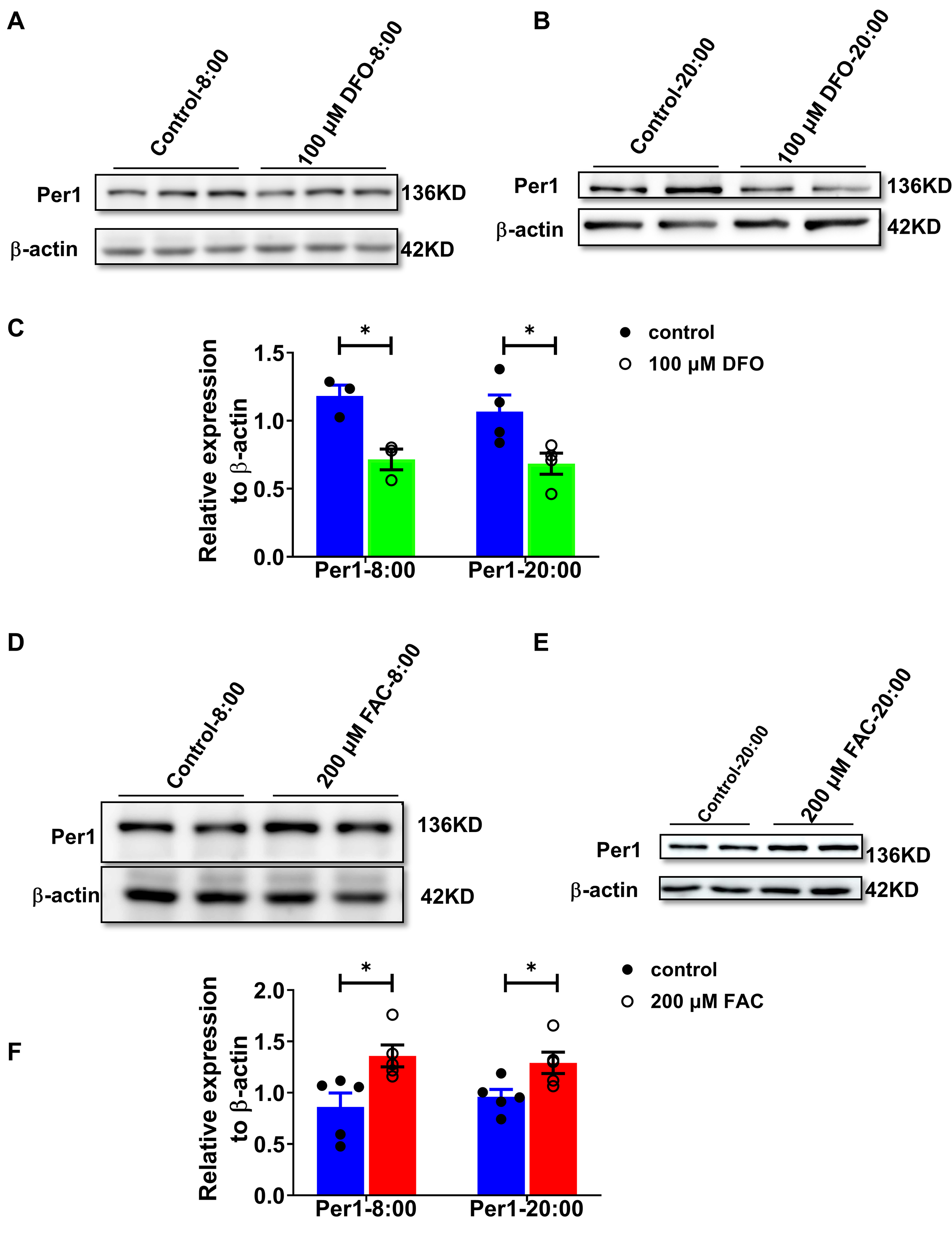

Supplement: Supplementary file 6 — Figure S6. [file CNS-30-e14592-s005.tif]
