# Supplementary material for: Cellular iron depletion enhances behavioral rhythm by limiting brain Per1 expression in mice
Source: CNS Neurosci Ther. 2024 Feb 22;30(2):e14592. doi: 10.1111/cns.14592 (PMC10883092; doi:10.1111/cns.14592)

Full unedited blot for Figure 1D

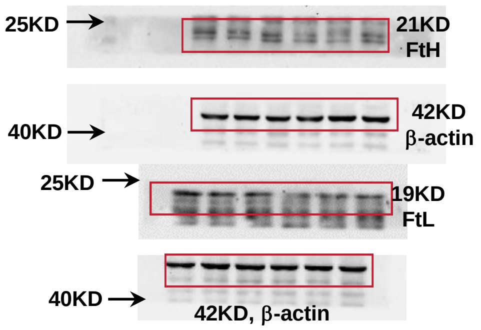

Full unedited blot for Figure 2F

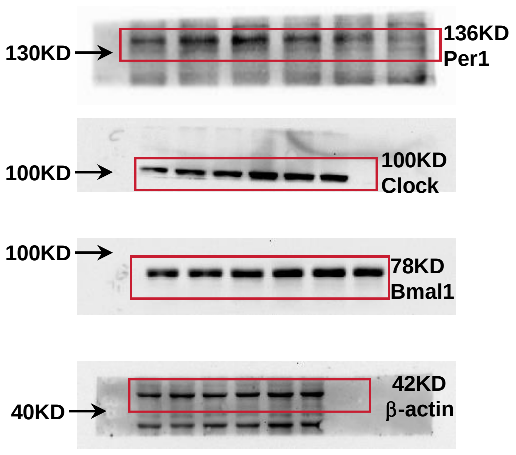

Full unedited blot for Figure 2H

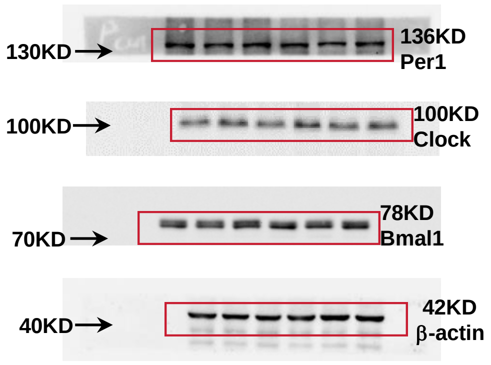

Full unedited blot for Figure 3B

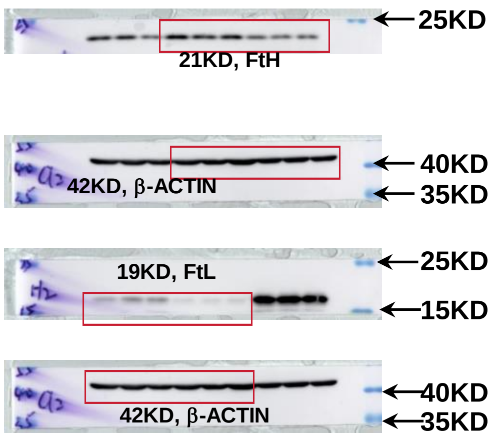

Full unedited blot for Figure 3D

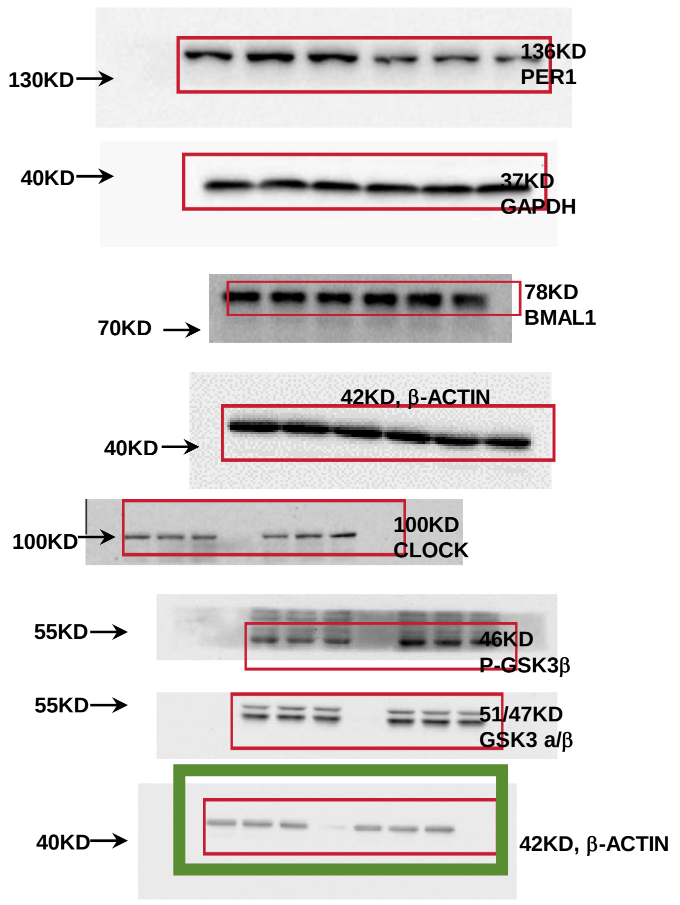

Full unedited blot for Figure 3G

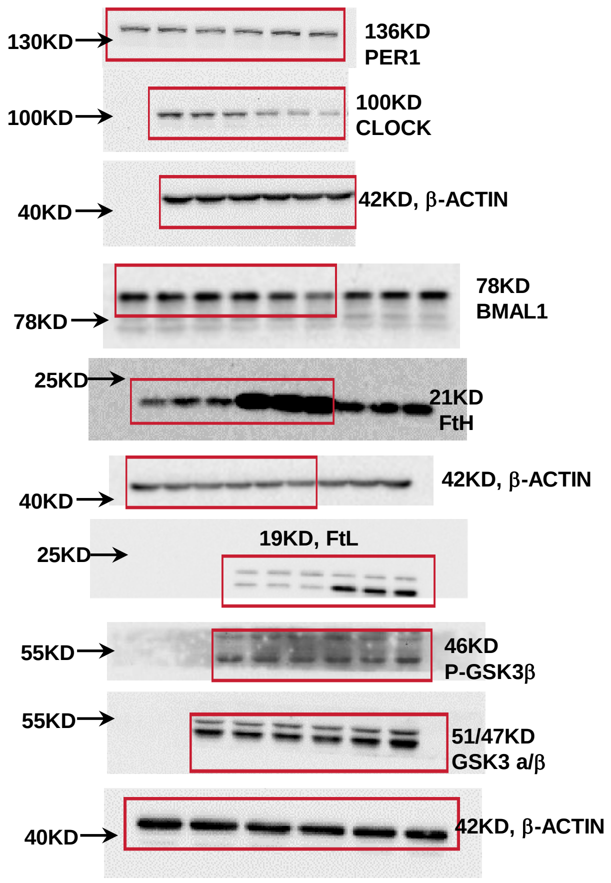

Full unedited blot for Figure 4A

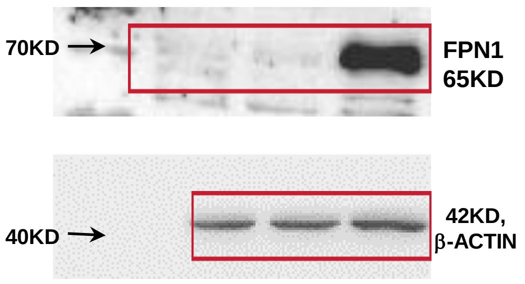

Full unedited blot for Figure 4C

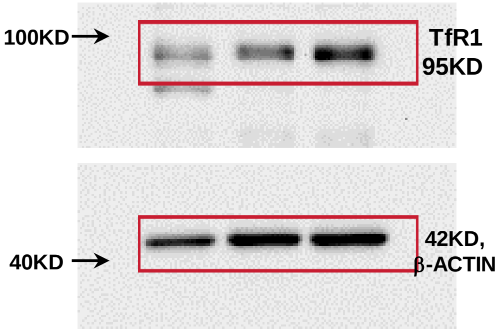

Full unedited blot for Figure 4E

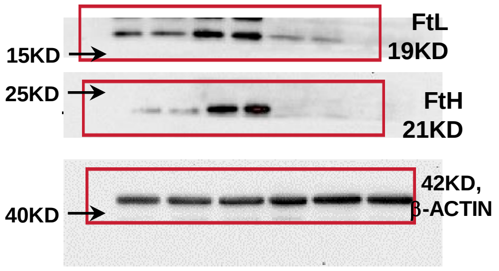

Full unedited blot for Figure 5A

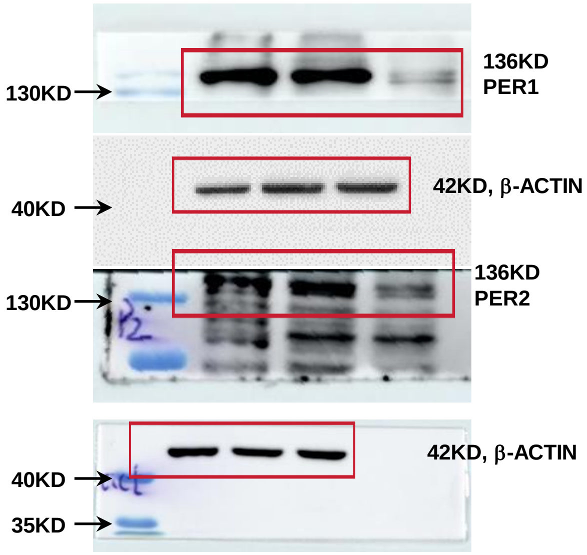

Full unedited blot for Figure 5C

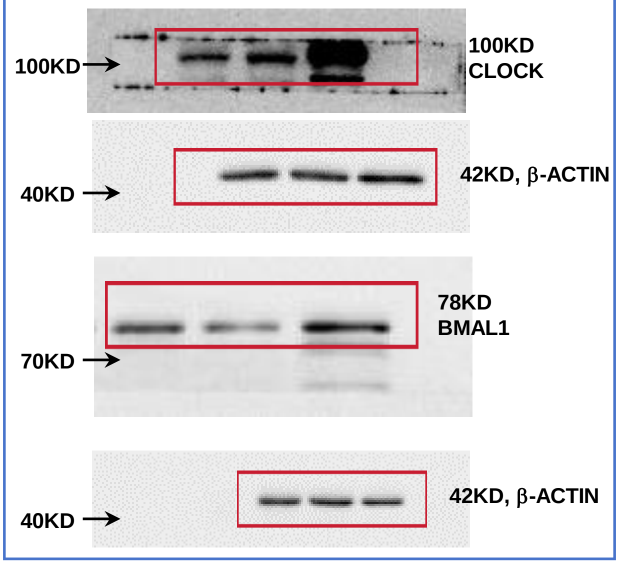

Full unedited blot for Figure 5E

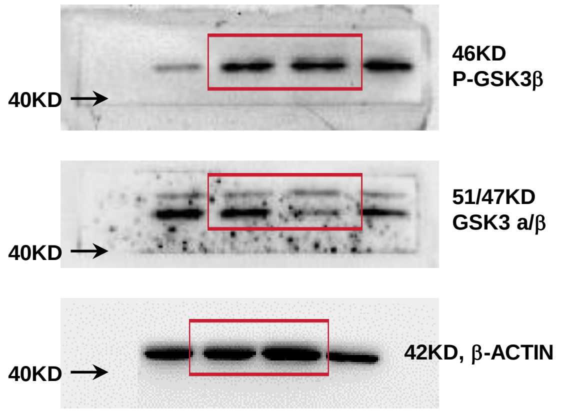

Full unedited blot for Figure 5G

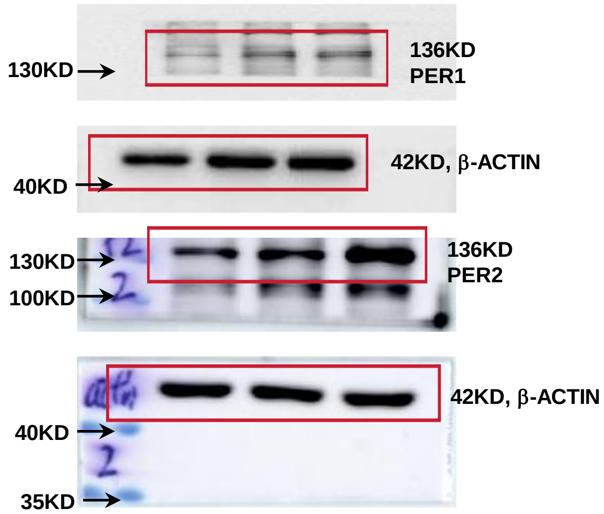

Full unedited blot for Figure 5I

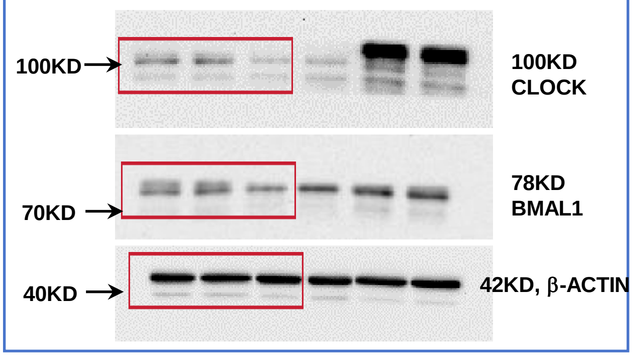

Full unedited blot for Figure 5K

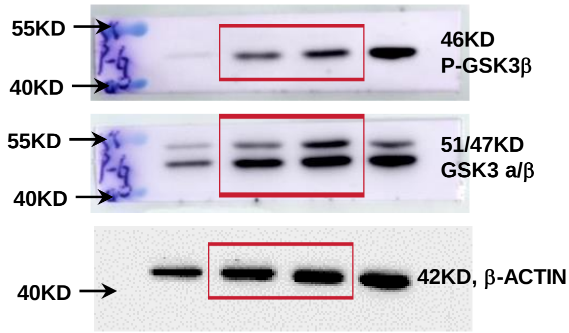

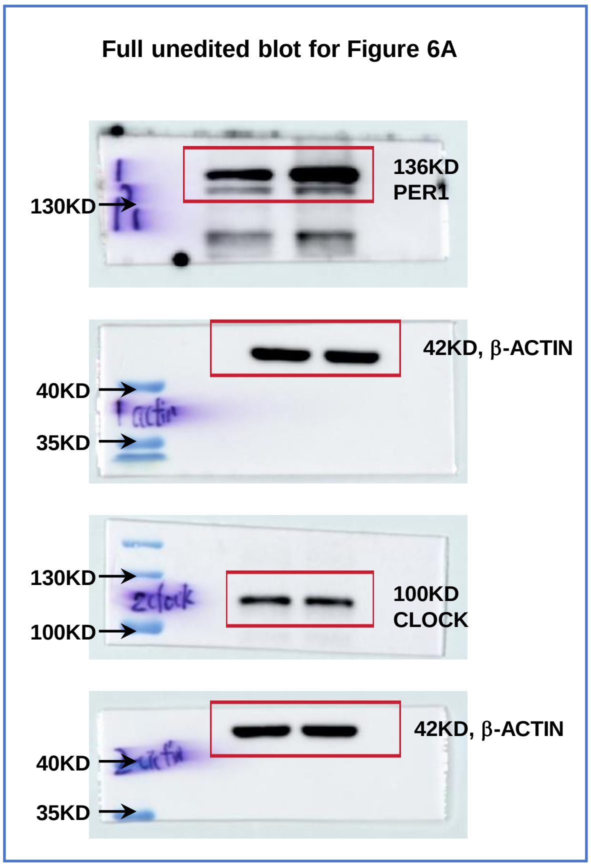
Full unedited blot for Figure 6A
130KD
136KD
PER1
42KD, β-ACTIN
40KD
35KD
130KD
100KD
100KD
CLOCK
42KD, β-ACTIN
40KD
35KD

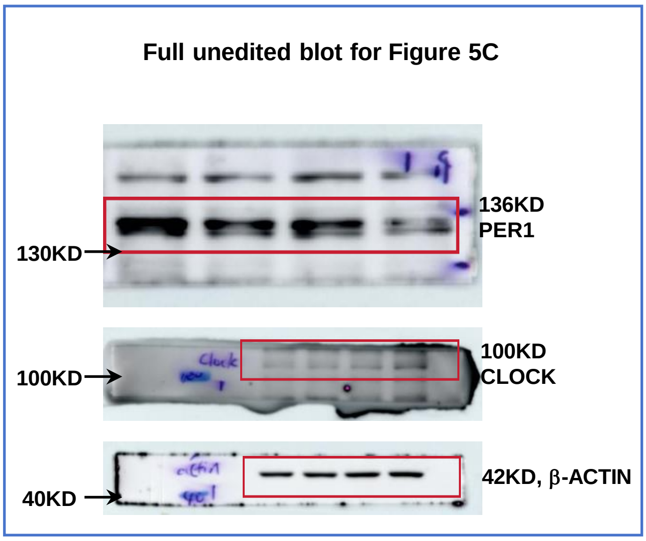
Full unedited blot for Figure 5C
136KD
PER1
130KD
100KD
100KD
CLOCK
42KD, β-ACTIN
40KD

Full unedited blot for Figure S3B

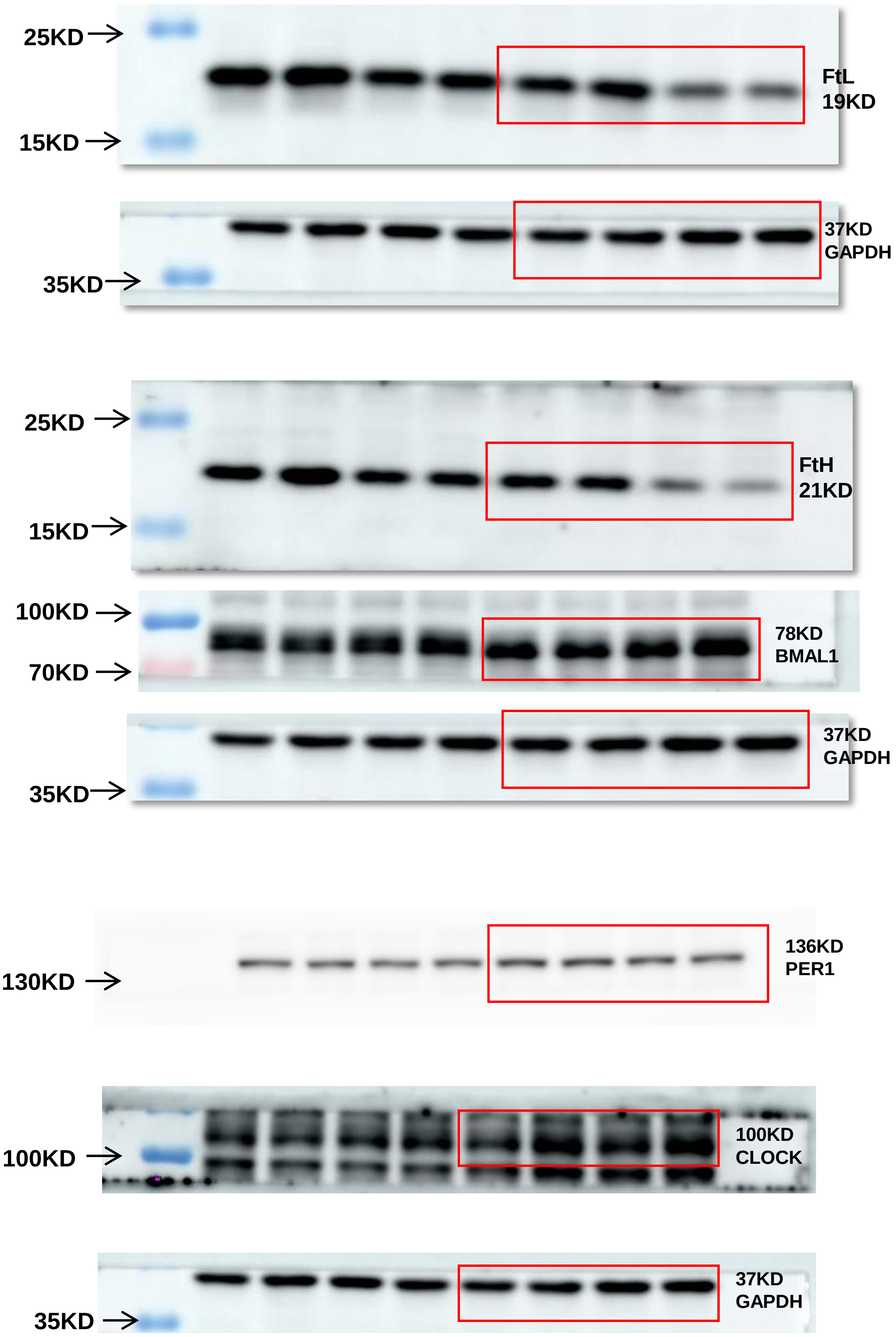

Full unedited blot for Figure S3E

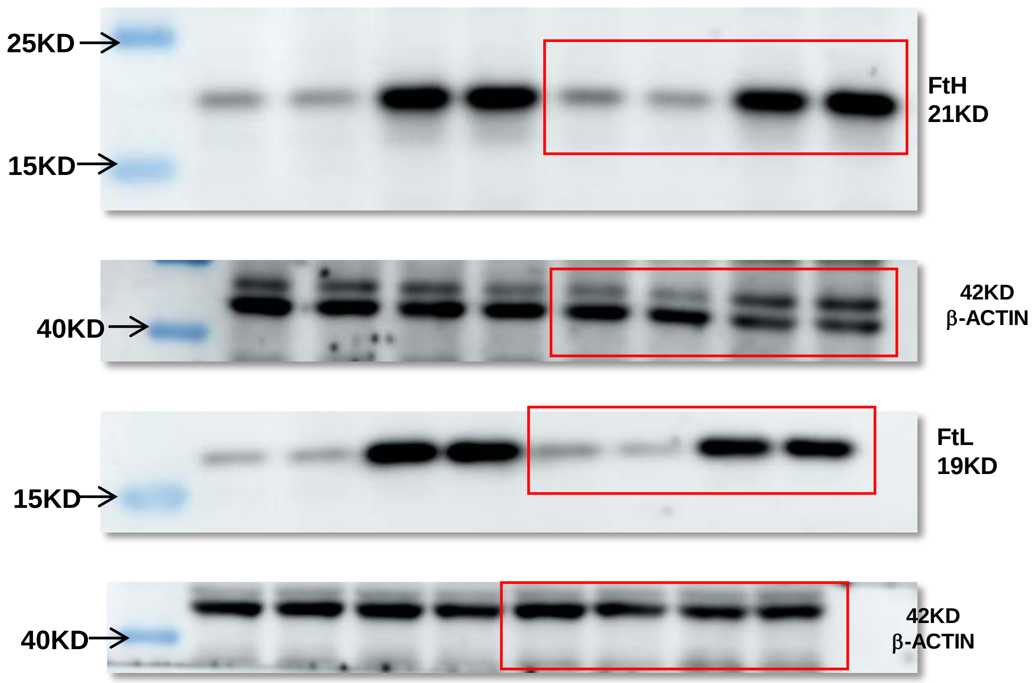

Full unedited blot for Figure S3G

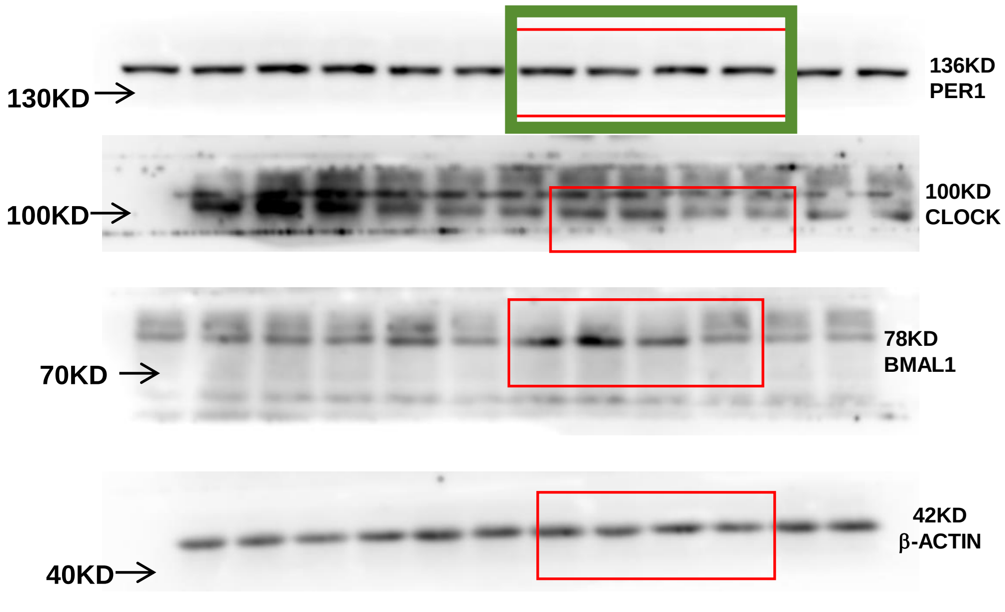

Full unedited blot for Figure S5C

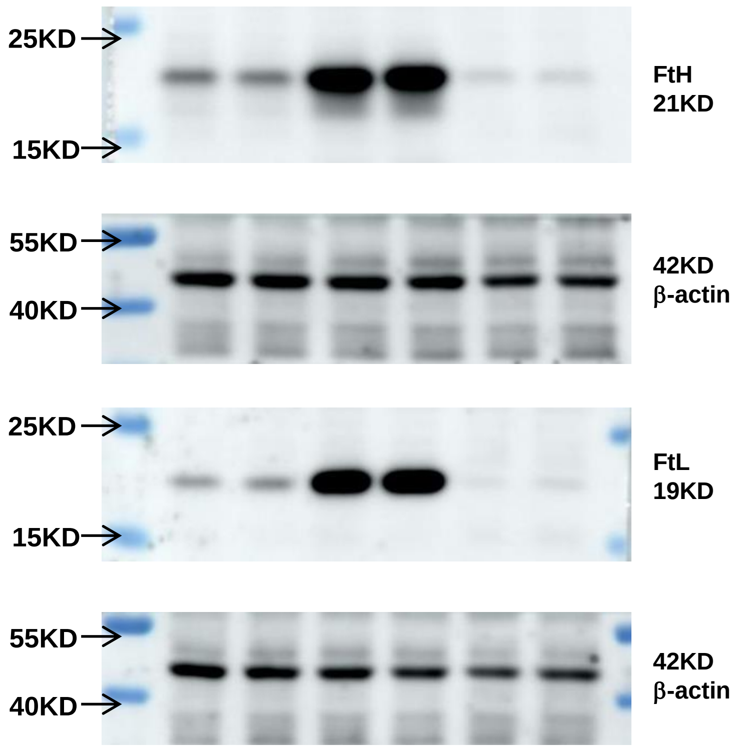

Full unedited blot for Figure S5D

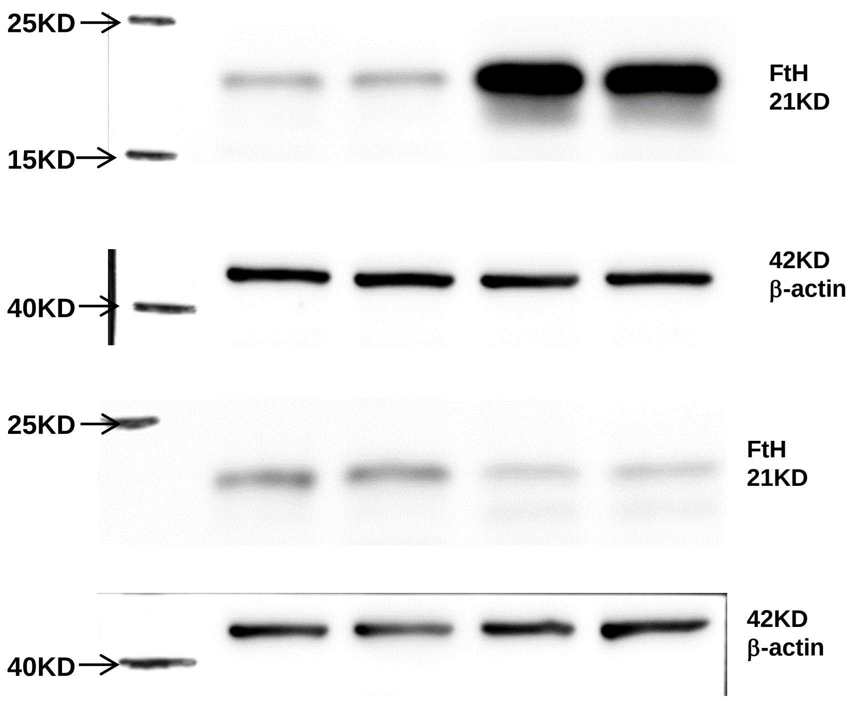

Full unedited blot for Figure S6A

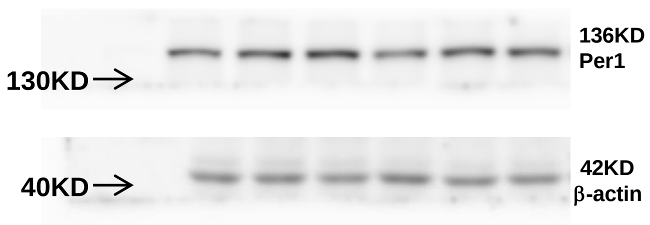

Full unedited blot for Figure S6B

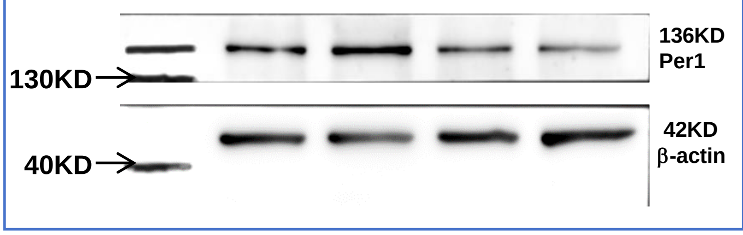

Full unedited blot for Figure S6D

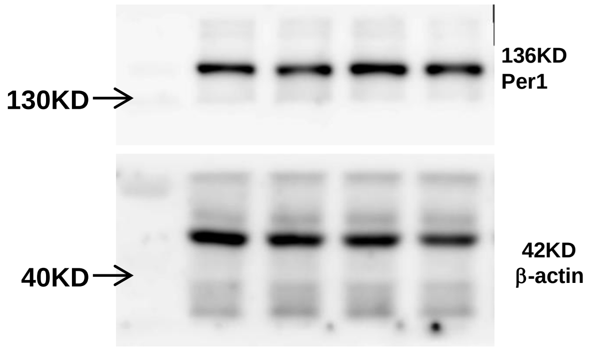

Full unedited blot for Figure S6E

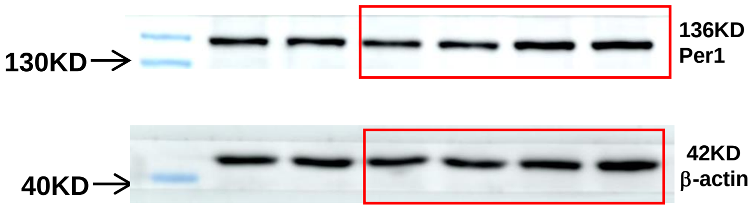

Supplement: Supplementary file 7 — Data S1. [file CNS-30-e14592-s007.pdf]
