# Supplementary material for: Cellular iron depletion enhances behavioral rhythm by limiting brain Per1 expression in mice
Source: CNS Neurosci Ther. 2024 Feb 22;30(2):e14592. doi: 10.1111/cns.14592 (PMC10883092; doi:10.1111/cns.14592)
Supplement: Supplementary file 8 — Data S2. [file CNS-30-e14592-s006.docx]

**Supporting information:**

**Figure S1. Rhythmic expression of *BMAL1*, *CLOCK*, *PER1* and *CRY1* mRNA in U251 cells.**

(**A**)–(**D**): U251 cells were harvested and the total RNA was extracted at 0, 4, 8, 12, 16, and 20 h. Next, *BMAL1*, *CLOCK*, *PER1* and *CRY1* mRNA levels were assessed by the quantitative RT-PCR. The data are expressed as the mean ± SEM, *n* = 3. Statistical analysis was performed using a two-tailed Student’s t-test. **P* < 0.05, ***P* < 0.01, ****P* < 0.001 compared to the control group (0 h).

**Figure S2. Cell viability assay in U251 cells treated with different concentrations of FAC or DFO.**

(**A**) the cell viability after treatment with the indicated concentrations of FAC. (**B**) the cell viability after treatment with the indicated concentrations of DFO. The data are expressed as the mean ± SEM, *n*=6. Statistical analysis was performed using a two-tailed Student’s t-test. **P* < 0.05, #*P* < 0.01, vs. the control group.

**Figure S3.** **Altered expression of PER1, CLOCK and BMAL1 in U251 cells treated with 50 μM DFO or 100 μM FAC at 8 pm.**

(**A**) Schematic illustration of the treatment of cells with DFO or FAC; the cells were collected and assayed at 8 am. (**B**) Representative western blot analysis results for FtL, FtH, BMAL1, PER1 and CLOCK in U251 cells treated with 50 μM DFO. (**C-D**) Statistical analysis of the results of the experiment shown in panel B. The data were quantified after normalizing to the respective GAPDH expression. (**E**) Representative western blot analysis results for FtH and FtL in U251 cells treated with 100 μM FAC for 24 h. (**F**) Statistical analysis of FtH and FtL compared to the control group after normalizing to the respective β-ACTIN expression. (**G**) Protein levels of PER1, CLOCK and BMAL1, as detected by western blot analysis in control and FAC-treated U251 cells. (**I**) Quantitative results of PER1, CLOCK and BMAL1 proteins. The data were quantified after normalizing to β-ACTIN expression. The data are presented as the mean ± SEM, *n* = 3. Statistical analysis was performed using a two-tailed Student’s t-test; **P* < 0.05, ***P* < 0.01, ****P* < 0.001compared to the control group.

**Figure S4. Rhythmic expression of *Bmal1, Clock, Per1 and Cry1* mRNA in MA-c cells.**

(A-D): MA-c cells were harvested and the total RNA was extracted at 0, 4, 8, 12, 16, and 20 h. Next, *Bmal1* (A), *Clock* (B), *Per1* (C) and *Cry1* (D) mRNA levels were assessed by quantitative RT-PCR. The data are expressed as the mean ± SEM, n = 3. Statistical analysis was performed using a two-tailed Student’s t-test. **P* < 0.05, ***P* < 0.01, ***vs. *P* < 0.001, vs. the 8:00 group.

**Figure S5. Cell viability assay (upper panel) and validation of FtH and FtL expression (lower panel) in MA-c cells treated with different concentrations of FAC or DFO.**

(A) Cell viability after treatment with the indicated concentrations of DFO. (B) Cell viability after treatment with the indicated concentrations of FAC. The data are presented as the mean ± SEM. Statistical analysis was conducted using a two-tailed Student’s t-test. **P* < 0.05 compared to the control group. (C-D) Representative western blot analysis results for FtH and FtL in MA-c cells treated with 100 μM DFO or 200 μM FAC. The cells were harvested and analyzed at 8:00 (C) or 20:00 (D).

**Figure S6. Altered expression of Per1 in MA-c cells treated with 100 μM DFO or 200 μM FAC at 8:00 am or 20:00 pm.**

(A-B) Protein levels of Per1 at 8:00 (A) or 20:00 (B) in MA-c cells treated with 100 μM DFO, as detected by western blot analysis. (C) Statistical analysis of the results of the experiments shown in panels A and B. (D-E) Representative western blot analysis results for Per1 in MA-c cells treated with 200 μM FAC, at 8:00 (D) or at 20:00 (E). Panel F shows the statistical analysis of the experiment shown in panels D and E. The data were quantified after normalizing to the respective β-actin expression. The data are expressed as the mean ± SEM. Statistical analysis was performed using a two-tailed Student’s t-test. **P* < 0.05, vs. the control group.
